# Explaining the influence of non‐shared environment (NSE) on symptoms of behaviour problems from preschool to adulthood: mind the missing NSE gap

**DOI:** 10.1111/jcpp.13729

**Published:** 2022-11-27

**Authors:** Agnieszka Gidziela, Margherita Malanchini, Kaili Rimfeld, Andrew McMillan, Angelica Ronald, Essi Viding, Alison Pike, Kathryn Asbury, Thalia C. Eley, Sophie von Stumm, Robert Plomin

**Affiliations:** ^1^ School of Biological and Chemical Sciences Queen Mary University of London London UK; ^2^ Social, Genetic & Developmental Psychiatry Centre, Institute of Psychiatry, Psychology & Neuroscience King's College London London UK; ^3^ Department of Psychology Royal Holloway University of London Egham UK; ^4^ Department of Psychological Sciences Birkbeck University of London London UK; ^5^ Division of Psychology and Language Sciences University College London London UK; ^6^ School of Psychology University of Sussex Brighton UK; ^7^ Department of Education University of York York UK

**Keywords:** Behaviour problem symptoms, non‐shared environment, twin study

## Abstract

**Background:**

Individual differences in symptoms of behaviour problems in childhood and adolescence are not primarily due to nature *or* nurture – another substantial source of variance is non‐shared environment (NSE). However, few specific environmental factors have been found to account for these NSE estimates. This creates a ‘missing NSE' gap analogous to the ‘missing heritability’ gap, which refers to the shortfall in identifying DNA differences responsible for heritability. We assessed the extent to which variance in behaviour problem symptoms during the first two decades of life can be accounted for by measured NSE effects after controlling for genetics and shared environment.

**Methods:**

The sample included 4,039 pairs of twins in the Twins Early Development Study whose environments and symptoms of behaviour problems were assessed in preschool, childhood, adolescence and early adulthood via parent, teacher and self‐reports. Twin‐specific environments were assessed via parent‐reports, including early life adversity, parental feelings, parental discipline and classroom environment. Multivariate longitudinal twin model‐fitting was employed to estimate the variance in behaviour problem symptoms at each age that could be predicted by environmental measures at the previous age.

**Results:**

On average across childhood, adolescence and adulthood, parent‐rated NSE composite measures accounted for 3.4% of the reliable NSE variance (1.0% of the total variance) in parent‐rated, symptoms of behaviour problems, 0.5% (0.1%) in teacher‐rated symptoms and 0.9% (0.5%) in self‐rated symptoms after controlling for genetics, shared environment and error of measurement. Cumulatively across development, our parent‐rated NSE measures in preschool, childhood and adolescence predicted 4.7% of the NSE variance (2.0% of the total variance) in parent‐rated and 0.3% (0.2%) in self‐rated behaviour problem symptoms in adulthood.

**Conclusions:**

The missing NSE gap between variance explained by measured environments and total NSE variance is large. Home and classroom environments are more likely to influence behaviour problem symptoms via genetics than via NSE.

## Introduction

Symptoms of behaviour problems are characterised by abnormalities in behavioural, cognitive and adaptive functioning that often begin in childhood and persist throughout the life course (Kessler et al., 2005; Reef, van Meurs, Verhulst, & van der Ende, 2010). An important source of individual differences in symptoms of behaviour problems are non‐shared environmental (NSE) effects (Plomin, 2011; Plomin, Asbury, & Dunn, 2001; Plomin & Daniels, 1987). Shared environmental influences denote what is usually meant by the word *nurture* – environmental influences that make children growing up in the same family similar (Harris, 1998). NSE refers to residual environmental influences that do not contribute to similarity of family members. In other words, NSE effects are what makes siblings growing up in the same family environment different (Knopik, Neiderhiser, DeFries, & Plomin, 2017). Examples of NSE effects include differential treatment that the twins receive from parents, as well as differences in external environment, such as classroom or peer group environment.

The finding that NSE influences behaviour problem symptoms in childhood and adolescence, while genetic and shared environmental influences are modest, is one of the most important and consistently replicated findings from genetic research (Plomin, DeFries, Knopik, & Neiderhiser, 2016). The importance of NSE was first pointed out almost 50 years ago (Loehlin & Nichols, 2012), first reviewed in 1987 (Plomin & Daniels, 1987) and first popularised in 1998 (Harris, 1998). Yet, little progress has been made toward identifying specific NSE factors that predict symptoms of behaviour problems (Dunn & Plomin, 1990; Turkheimer & Waldron, 2000). In 2000, a meta‐analysis of 43 papers relating sibling differences in environmental measures to sibling differences in outcomes concluded that ‘measured non‐shared environmental variables do not account for a substantial portion of the non‐shared variability’ (Turkheimer & Waldron, 2000).

Turkheimer and Waldron's (2000) review suggested that research into identifying the drivers of NSE influences was off to a good start. Of the variance in sibling differences in behavioural adjustment, personality and cognitive traits, 1% could be attributed to family constellation (i.e. variables related to birth order and age differences between siblings), 2% to differential parenting behaviour, 2% to differential sibling interaction and 5% to differential peer or teacher interaction (Turkheimer & Waldron, 2000). Moreover, these effects were largely independent and together they account for 13% of the between‐sibling variance (Turkheimer & Waldron, 2000). However, estimates of NSE influence are halved in designs that controlled for genetics (Turkheimer & Waldron, 2000). Another issue is that Turkheimer and Waldron's (2000) meta‐analysis focused on variance in sibling differences, not total variance in behavioural adjustment, personality and cognitive traits. Translating the effect sizes for sibling differences to total variance estimates suggests that the estimates of NSE effects would be at least halved again when NSE variance is 0.50.

Two genetically sensitive designs have been used to disentangle genetic and environmental sources of sibling differences: The monozygotic (MZ) twin differences design and the multivariate genetic design (Martin & Eaves, 1977; Rovine, 1994). The MZ differences design involves correlating measured environmental differences within pairs of MZ twins with MZ differences in behaviour problem symptoms. This design captures NSE influence because MZ twins reared together are identical in terms of inherited DNA differences and shared environmental influences, so all their differences are due to NSE (Vitaro, Brendgen, & Arseneault, 2009). The first MZ differences study (Pike, Reiss, Hetherington, & Plomin, 1996) was part of the Nonshared Environment and Adolescent Development (NEAD) study, a longitudinal study of 720 families including twins and adopted children aimed at exploring the NSE effects on development of adolescent behaviour and psychopathology (Neiderhiser, Reiss, & Hetherington, 2007; Reiss et al., 1994; Reiss, Neiderhiser, Hetherington, & Plomin, 2000). The MZ differences study found moderate correlations between MZ differences in parental negativity and MZ differences in adolescent depression and antisocial behaviour (Pike, Reiss, et al., 1996).

Monozygotic differences studies have consistently reported low‐to‐moderate correlations between parenting style and behaviour problem symptoms. For example, MZ twin differences in maternal negativity correlated 0.49 and 0.17 with differences in antisocial behaviour at age 5 as rated by mothers and teachers, respectively (Caspi et al., 2004). Subsequently, these findings were replicated in a sample of 7‐year‐olds, by correlating MZ twin differences in negative parental discipline with differences in conduct problems and callous–unemotional traits, which yielded estimates of 0.46 and 0.27 for parent ratings and 0.12 and 0.07 for teacher ratings, respectively (Viding, Fontaine, Oliver, & Plomin, 2009).

Multivariate genetic analysis is better suited than the MZ differences analysis to answer the question of how much total variance in behaviour problem symptoms can be predicted by measured environments (Pike, McGuire, Hetherington, Reiss, & Plomin, 1996). Analogous to univariate genetic analysis that decomposes variance in a trait into genetic and environmental components of variance, multivariate genetic analysis decomposes the covariance between two traits – in this case, the covariance between an environmental measure and a measure of behaviour problems – into genetic, shared environmental and NSE components of covariance (Knopik et al., 2017).

The first multivariate genetic analysis of this type investigated child‐specific family environment measures and behaviour problem symptoms in 719 same‐sex pairs of adolescent siblings aged 10–18 years (Pike, McGuire, et al., 1996). A multi‐informant composite index of maternal negativity toward their child as rated by the mother, father and sibling correlated phenotypically 0.33 with a composite measure of the target child's depressive symptoms. Squaring the correlation of 0.33 indicated that 11% of the total variance in depressive symptoms could be predicted by maternal negativity.

Pike, McGuire, et al. (1996) found that NSE effects explained 1.2% of the reliable variance in depressive symptoms. Shared environment also explained 1.2% of variance, and genetic effects accounted for 17.6%. The reason why these estimates sum to 20%, greatly exceeding the 11% of total variance explained phenotypically by the measure of maternal negativity, is that the genetic (*a*), shared environmental (*c*) and NSE (*e*) paths from maternal negativity explain *reliable* variance in depressive symptoms. Error of measurement of the total variance in depressive symptoms is included in the *a, c* and *e* residual estimates.

Another multivariate twin study conducted using a sample of 808 same‐sex 11‐year‐old twin pairs from the Minnesota Twin Family Study reported findings consistent with those from the NEAD study (Burt, Krueger, McGue, & Iacono, 2003). A multi‐informant measure of parent–child conflict was found to explain 1% of the total variance in externalising disorders via NSE, with 20% accounted for by genetics and 12% by shared environment. Modest NSE prediction was also reported in a multivariate twin study involving 1,314 adolescent twin pairs from the Twin study of CHild and Adolescent Development (TCHAD), where parental criticism predicted <1% of the total variance in antisocial behaviour in boys and 0.4% in girls via NSE (Narusyte, Andershed, Neiderhiser, & Lichtenstein, 2007). In contrast, genetics accounted for 12% in boys and 18% in girls.

The current research follows through on three issues raised in the NEAD reports (Pike, McGuire, et al., 1996; Pike, Reiss, et al., 1996). First, rather than limiting the analysis to contemporaneous assessments of environment and behaviour problems symptoms, the present study uses a longitudinal twin design to systematically assess the extent to which environmental measures at one age can predict symptoms of behaviour problems at a later age via NSE after controlling for genetics and shared environment. Although this longitudinal approach embedded in a multivariate genetic design provides some purchase on causal inference, our goal here was prediction rather than addressing the complex issue of causality (Plomin & von Stumm, 2022). Second, instead of analysing individual environmental measures, our analyses assess the effect of multiple environmental measures on symptoms of behaviour problems. For that purpose, we created the multi‐environment composites that included measures of early life adversity, parental feelings and discipline and classroom environment. Third, we compare results for same‐rater (i.e. parent, teacher and self‐reports) and cross‐rater analyses to test for rater effects in prediction of behaviour problem symptoms.

In summary, the present study tested the longitudinal NSE prediction of behaviour problem symptoms as rated by parents, teachers and the twins themselves from parent‐rated environmental measures at earlier ages. We predicted behaviour problem symptoms in childhood at ages 7 and 9 from environmental measures in preschool (ages 3 and 4), behaviour problem symptoms in adolescence (ages 12 and 16) from environmental measures in childhood and behaviour problem symptoms in adulthood (age 21) from environmental measures in adolescence. We also investigated the extent to which symptoms of behaviour problems in adulthood are predicted cumulatively from NSE‐related environmental processes in preschool, childhood and adolescence.

## Methods

Our hypotheses and analyses were preregistered with the Open Science Framework (OSF; https://osf.io/rbv9q) prior to analysing the data. Our detailed hypotheses are listed in Appendix S1. Our analysis scripts are available on the OSF page and https://github.com/CoDEresearchlab/NSE_BP.

### Sample

Our sampling frame consisted of twins born in England and Wales between 1994 and 1996 who have been enrolled in the Twins Early Development Study (TEDS; Rimfeld et al., 2019).The present analyses included up to 4,039 pairs of twins with requisite environmental and behaviour problem data from infancy to early adulthood. Details of the sample and its representativeness are provided in Appendix S2 and Table S1.

### Measures

#### Environmental measures

We selected parent‐reported environmental measures for which twins in the same family could have different scores such as twin‐specific parenting, in contrast to family‐general measures such as parental education for which both twins have the same score, and which cannot be used in analyses of NSE. However, such ‘twin‐specific’ environmental measures do not assess completely different experiences of twins in a family. That is, twin correlations for such measures are often substantial, this covariance is included in the shared environment component in multivariate genetic analysis so that only the twin‐specific component is ascribed to NSE. Initially, measures included virtually all environmental items and scales available in TEDS data dictionary (https://www.teds.ac.uk/datadictionary/home.htm). We grouped the environmental measures in three age groups: preschool (ages 3 and 4), childhood (ages 7 and 9) and adolescence (ages 12 and 16).

As explained in Appendix S3, we reduced the hundreds of twin‐specific environmental items available in the TEDS data dictionary at each age to a single ‘poly‐E' composite after excluding measures with low correlations with behaviour problem symptoms at the subsequent developmental stage (cut‐off = 0.20, determined based on the distribution of correlations as illustrated in Figure S1). We also excluded highly correlated environmental measures. This criterion was applied as we created a ‘poly‐E' composite at each age using a penalised regression elastic net regularisation with hold‐out sample tests of prediction accuracy. This procedure overcomes problems of multicollinearity as well as overfitting (Allegrini et al., 2020; Gidziela et al., 2022; Zou & Hastie, 2005). The poly‐E composites included measures of early life adversity (aka environmental risk; Cox, Holden, & Sagovsky, 1987; Matheny Jr, Wachs, Ludwig, & Phillips, 1995), parental feelings and discipline (Deater‐Deckard, 1998) and classroom environment (Ainley & Bourke, 1992). For details of the construction of the poly‐E composites, see Appendix S4. Environmental variables surviving the selection process are listed and described in Table S2.

#### Behaviour problem measures

Hyperactivity‐inattention, conduct problems, emotional problems and peer relationship problems were assessed using the Preschool Behaviour Questionnaire (PBQ; Behar, 1977) at age 3 and Strengths and Difficulties Questionnaire (SDQ; Goodman, 2001) from age 4 to age 21. The four scales were combined in preschool (ages 3 and 4), childhood (ages 7 and 9), adolescence (ages 12 and 16) and adulthood (age 21) as rated by parents (ages 3–21), by teachers (ages 7–12) and by the twins (ages 9–21). For each of the four scales and three raters, mean scores were calculated across ages in childhood and in adolescence or set to missing if more than half of the data was missing. This data reduction resulted in 36 behaviour problem symptoms variables for the four scales, three ages and three raters, as summarised in Figure S2.

### Analyses

We used univariate twin model‐fitting analyses to estimate components of variance for the 36 behaviour problem symptoms variables. Bivariate twin model‐fitting (Cholesky decomposition) analysis (see Appendix S5 and Figure S3) was used to estimate the variance in behaviour problem symptoms variables at one developmental stage (e.g. childhood) predicted by the poly‐E composite at the previous stage (e.g. preschool). Analyses were conducted for same‐rater comparisons (i.e. predicting parent‐rated behaviour problem symptoms from parent‐rated poly‐E composites), as well as for cross‐rater comparisons (i.e. predicting teacher and self‐rated behaviour problem symptoms from parent‐rated poly‐E composites). Multivariate twin model‐fitting analysis was also used to estimate the variance in parent and self‐rated behaviour problem symptoms at age 21 predicted cumulatively by parent‐rated poly‐E composites from preschool, childhood and adolescence (Figure S3). For details of these twin analyses, see Appendix S5.

We compared the bivariate twin model‐fitting results to results from analyses using the MZ differences design. As explained in Appendix S6, we created relative difference scores for MZ twins for the poly‐E variables and correlated them with MZ difference scores for the behaviour problem symptoms variables. As an alternative to MZ difference scores, we also created indices of within‐pair differences for the poly‐E and behaviour problem variables from the standardised residuals after regressing Twin 1's scores on Twin 2's scores. We correlated these residualised scores and simple MZ difference scores with behaviour problem symptoms of individuals to estimate the NSE effect on variation in behaviour problem symptoms.

## Results

We present results in four sections. The first section summarises estimates of the NSE, genetic and shared environmental variance for behaviour problem symptoms and poly‐E composites over development. The second section describes contemporaneous as well as longitudinal phenotypic correlations between poly‐E measures and behaviour problem symptoms. The third section describes the prediction of behaviour problem symptoms at each age from environmental measures at the previous age. The fourth section addresses the cumulative prediction of behaviour problem symptoms in adulthood from environmental measures in preschool, childhood and adolescence. The fifth section outlines the result of MZ differences and residualised scores analyses.

### Univariate twin analyses

**Figure 1:**
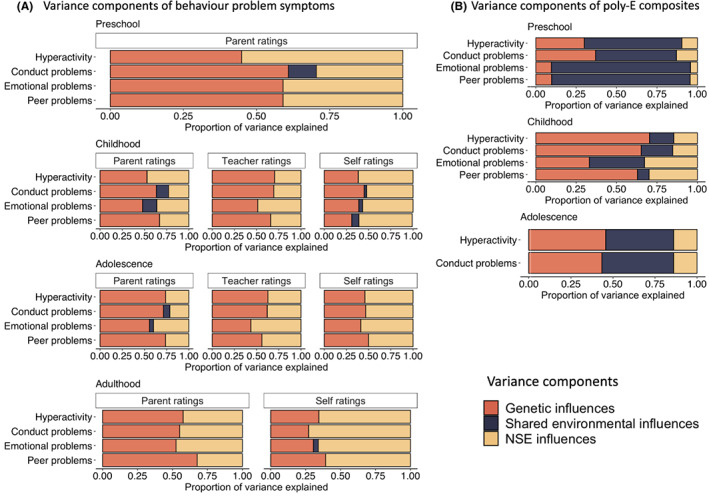
Genetic, shared environmental and non‐shared environmental (NSE) components of variance in behaviour problem symptoms (panel A) and poly‐E composites (i.e. environmental measures; panel B) across development, rated by parents, teacher and the twins themselves. *Note*. Different poly‐E composites were created for each behaviour problem measure, that is hyperactivity, conduct problems, emotional problems and peer problems. Results for poly‐E composites for emotional problems and peer problems in adolescence are not included due to weak correlations with E measures (*r* < .20), meaning that they fell below our criterion for inclusion in poly‐E composites

Figure 1 illustrates the NSE, genetic and shared environmental components of variance from the univariate twin model fitting of behaviour problem symptoms (panel A) and poly‐E composites (panel B). These estimates, along with 95% confidence intervals are presented in Table S3 for the total sample. Tables S4 and S5 show that results are not significantly different between males and females, as shown by the overlapping 95% confidence intervals.

#### Behaviour problem symptoms

For parent‐rated behaviour problem symptoms, NSE influences plus error of measurement on average accounted for about a third (37%) of the variance (43% for hyperactivity, 30% for conduct problems, 41% for emotional problems and 33% for peer problems), with three quarters accounted for by genetic influences (60%) and with little to no shared environmental contribution (3%; Figure 1A). For teacher‐rated behaviour problem symptoms, the mean NSE estimate was 40% and ranged from 33% for hyperactivity to 53% for emotional problems, while the rest of the variance was accounted for by genetic influences (60%). The largest average NSE estimates across developmental stages were observed for self‐rated symptoms of behaviour problems, 59% on average, ranging from 56% for peer problems to 61% for emotional problems, with genetics being the second largest contributing factor (39%) and with little shared environmental influences (2%). Across all four behaviour problems measures, NSE accounted for more variance in adulthood (54%) compared to preschool (42%), childhood (41%) and adolescence (42%).

#### Poly‐E composites

As seen in Figure 1B, across ages, NSE accounted for much less of the variance in the poly‐E composites as compared to behaviour problem symptoms (Figure 1A). In the preschool years, NSE accounted for only 8% of the variance in poly‐E composites, with most of the variance explained by shared environmental influences (71%) and with a moderate contribution of genetics (22%). In childhood, NSE influences explained 23% of the variance, with genetic influences accounting for 58% and shared environment for 19%. In adolescence, NSE accounted for 14% of the variance, with similar contributions from genetics (45%) and shared environment (41%).

### Phenotypic correlations

Although we focus on the longitudinal prediction of behaviour problem symptoms from earlier environmental measures, contemporaneous correlations between poly‐E composites and symptoms of behaviour problems (i.e. correlations between poly‐E composites in preschool, childhood and adolescence and behaviour problem symptoms at the same age) are shown in Figure S5. As expected, these contemporaneous correlations are greater than the longitudinal correlations between behaviour problem symptoms and earlier environmental measures.

The average contemporaneous correlations between poly‐E composites and parent‐rated behaviour problem symptoms were 0.38 in preschool, 0.55 in childhood and 0.43 in adolescence. In contrast, the mean longitudinal correlations between preschool, childhood and adolescence poly‐E measures and parent‐rated behaviour problem symptoms at subsequent developmental stages (i.e. childhood, adolescence and adulthood) were 0.31, 0.41 and 0.25, respectively.

### Bivariate twin analyses

Table 1 presents the proportions of variance in behaviour problem symptoms in childhood, adolescence and adulthood predicted by environmental measures (poly‐E composites) at the previous age. Figure S4 shows the NSE path analytic results underlying Table 1. Table S6 presents the full model‐fitting results for genetic, shared environmental and NSE components of covariance, as well as 95% confidence intervals for path estimates for the total sample. Tables S7 and S8 contain results separately for males and females, which are highly similar.

**Table 1:** Non‐shared environmental (NSE), genetic and shared environmental results of the bivariate Cholesky model of poly‐E composites (i.e. environmental measures) in preschool, childhood and adolescence predicting variance in measures of behaviour problem symptoms in subsequent developmental stages

Behaviour problem measure	Rater	Developmental stage	% of variance explained via NSE		% of variance explained via genetics		% of variance explained via shared environment	
			% of NSE variance	% of total variance	% of genetic variance	% of total variance	% of shared environmental variance	% of total variance
Hyperactivity	Parent	Childhood	2.22	1.06	32.45	16.05	100.00	1.33
Conduct problems			2.34	0.54	34.15	21.45	19.67	2.70
Emotional problems			0.46	0.17	1.10	0.53	52.79	8.32
Peer problems			0.96	0.32	0.55	0.33	100.00	4.57
Hyperactivity	Teacher	Childhood	0.12	0.04	12.24	8.46	100.00	0.09
Conduct problems			0.01	0.00	12.24	8.40	100.00	0.02
Emotional problems			0.46	0.22	0.19	0.10	100.00	0.53
Peer problems			0.20	0.07	1.03	0.66	100.00	0.52
Hyperactivity	Self	Childhood	0.46	0.28	17.77	6.78	100.00	0.06
Conduct problems			0.48	0.25	16.32	7.36	62.26	1.63
Emotional problems			0.07	0.04	2.82	1.09	94.37	4.15
Peer problems			0.08	0.05	1.94	0.60	30.10	2.61
Hyperactivity	Parent	Adolescence	9.11	2.30	35.67	25.13	100.00	0.12
Conduct problems			6.02	1.27	29.61	20.87	7.56	0.54
Emotional problems			4.33	1.72	18.97	10.51	34.13	1.60
Peer problems			7.71	1.98	32.94	23.76	100.00	0.14
Hyperactivity	Teacher	Adolescence	2.04	0.75	13.62	8.49	99.99	0.00
Conduct problems			0.74	0.28	4.52	2.78	100.00	0.22
Emotional problems			0.05	0.03	7.83	3.35	100.00	0.51
Peer problems			0.19	0.08	17.56	9.74	100.00	0.00
Hyperactivity	Self	Adolescence	2.28	1.23	19.33	8.76	100.00	0.14
Conduct problems			2.96	1.59	12.10	5.38	100.00	1.45
Emotional problems			1.60	0.94	5.37	2.12	100.00	1.33
Peer problems			0.39	0.19	24.61	12.17	100.00	0.07
Hyperactivity	Parent	Adulthood	0.77	0.32	13.52	7.59	100.00	0.11
Conduct problems			0.32	0.14	20.27	11.07	100.00	0.31
Hyperactivity	Self	Adulthood	0.08	0.05	3.24	1.11	100.00	0.33
Conduct problems			0.07	0.05	14.36	3.88	100.00	0.03

#### Prediction of behaviour problem symptoms from poly‐E composites via NSE

Table 1 summarises the NSE results of Cholesky decomposition analysis of parent‐rated poly‐E composites and behaviour problem symptoms (parent, teacher and self‐rated). As shown in Figure S3, the Cholesky model decomposes the variance in behaviour problem symptoms into variance explained by the environmental measure and the rest of the variance independent of the environmental measure. For example, the NSE estimate for parent‐rated hyperactivity in childhood (i.e. the sum of squared paths e12 and e22) is 48%. The preschool poly‐E composite explains 2.2% of this NSE variance or 1.1% of the total variance. In other words, more than 98% of the total variance in childhood hyperactivity is not explained by NSE processes related to the poly‐E composite.

On average, poly‐E composites predicted 3.4% of the reliable NSE variance (1.0% of the total variance) in parent‐rated symptoms of behaviour problems, 0.5% (0.2%) in teacher‐rated symptoms and 0.9% (0.5%) in self‐rated symptoms. Poly‐E composites accounted for more variance in behaviour problem symptoms in adolescence (3.1% of the NSE variance or 1.0% of the total variance), than in childhood (0.7% or 0.3%) and in adulthood (0.3% or 0.1%). Similar proportions of NSE variance (or total variance) were accounted for in hyperactivity (2.1% or 0.8%), conduct problems (1.6% or 0.5%), emotional problems (1.2% or 0.5%) and peer problems (1.6% or 0.5%).

#### Prediction of behaviour problem symptoms from poly‐E composites via genetics

As presented in Table 1, genetics accounted for much more of the poly‐E prediction of behaviour problem symptoms. On average, genetic processes explained 13.7% of the total variance in parent ratings of symptoms of behaviour problems, 5.3% in teacher and 4.9% in self‐reports. Consistently higher prediction across developmental stages emerged for hyperactivity (10.3%) and conduct problems (10.2%) as compared to emotional (3.0%) and peer problems (7.9%). The mean proportion of total variance explained via genetics was higher in adolescence (11.1%) than in childhood (6.0%) and adulthood (5.9%).

#### Prediction of behaviour problem symptoms from poly‐E composites via shared environment

Table 1 also presents Cholesky results for parent, teacher and self‐rated behaviour problem symptoms as predicted by poly‐E composites via shared environment. In childhood and adolescence, the variance explained by poly‐E composites via shared environment was modest (2.2% and 0.5%, respectively). Shared environmental influences were not present in behaviour problem symptoms in adulthood.

### Multivariate twin analyses

Table 2 summarises results of Cholesky decomposition analysis predicting parent‐ and self‐rated hyperactivity and conduct problems in adulthood cumulatively from parent‐rated poly‐E composites in preschool, childhood and adolescence, via NSE, genetics and shared environment. Figure S6 shows the NSE path models summarised in Table 2. Table S9 includes the full model‐fitting results and confidence intervals. Results for emotional problems and peer problems are not included due to weak correlations with environmental measures (*r* < .20) that they fell below our criterion for inclusion in poly‐E composites.

**Table 2:** Non‐shared environmental (NSE), genetic and shared environmental results of the multivariate Cholesky model of poly‐E composites (i.e. environmental measures) in preschool, childhood and adolescence cumulatively predicting variance in hyperactivity and conduct problems in adulthood

Behaviour problem measure	Rater	Developmental stage	% of variance explained via NSE		% of variance explained via genetics		% of variance explained via shared environment	
			% of NSE variance	% of total variance	% of genetic variance	% of total variance	% of shared environmental variance	% of total variance
Hyperactivity	Parent	Adulthood	4.57	1.91	20.75	11.00	–	1.00
Conduct problems	Parent	Adulthood	4.85	2.17	20.75	11.00	–	1.00
Hyperactivity	Self	Adulthood	0.52	0.34	12.50	4.00	–	1.00
Conduct problems	Self	Adulthood	0.13	0.10	25.00	6.00	–	2.00

#### Cumulative (longitudinal) prediction via NSE

The NSE variance in parent‐rated hyperactivity in adulthood is 42%. Cumulatively, the poly‐E measures in preschool, childhood and adolescence predict 4.6% of this NSE variance, or 1.9% of the total variance in hyperactivity. On average, poly‐E composites cumulatively across development predicted 4.7% of the NSE variance (2.0% of the total variance) in parent‐rated and 0.3% (0.2%) in self‐rated symptoms of behaviour problems in adulthood. Similar proportions of the NSE variance were accounted for in conduct problems (2.5% or 1.1% of the total variance) and hyperactivity (2.5% or 110%).

#### Cumulative (longitudinal) prediction via genetics

Poly‐E composites cumulatively across development predicted 11.0% of the total variance in parent‐rated and 5.0% in self‐rated symptoms of behaviour problems in adulthood via genetics (Table 2). The poly‐E composites accounted for a similar proportion of variance in hyperactivity (7.5%) and conduct problems (8.5%).

#### Cumulative (longitudinal) prediction via shared environment

Table 2 also presents shared environmental results of the longitudinal multivariate Cholesky decomposition. Because no shared environmental variance was found for symptoms of behaviour problems in adulthood, shared environmental processes did not contribute to the prediction of behaviour problem symptoms in adulthood from poly‐E composites at earlier ages.

### Comparing results from MZ differences design and residualised scores

We compared our Cholesky results to those using the MZ differences design rather than the full twin model. In general, correlations between MZ poly‐E differences and MZ behaviour problem symptom differences (Figure S7) yielded similar NSE estimates as Cholesky decomposition, as illustrated in Figure S8. Results of the MZ differences analysis are described in Appendix S7. Figure S8 shows that NSE results obtained using the residualised scores approach are also similar to those obtained from MZ differences and Cholesky analyses. Figure S9 presents correlations between these residualised poly‐E and behaviour problem measures.

## Discussion

Our attempt to assess the extent to which parent‐rated environmental measures taken together predict NSE effects on behaviour problem symptoms during the first two decades of life revealed the large ‘missing NSE' gap between the variance explained by measured environments and the NSE variance of behaviour problem symptoms estimated from twin studies (Turkheimer, 2011).

We were especially interested in the long‐term ability of parent ratings of earlier environments to predict NSE variance in adult self‐reports of behaviour problem symptoms because many studies focus on predicting adult self‐reports of behaviour from parents' ratings of early environments. Cumulatively across development, our parent‐rated poly‐E measures in preschool, childhood and adolescence predicted only 0.3% of the reliable NSE variance in self‐rated symptoms of behaviour problems in adulthood. In contrast, parent‐rated poly‐E measures cumulatively accounted for 4.7% of the NSE variance in parent‐rated symptoms of behaviour problems in adulthood. These predictions of parent‐rated symptoms are much greater than predictions of self‐rated symptoms presumably because the same rater (the parent) rated both the poly‐E measures and the symptoms. All of these predictions are weaker when they are converted to the total variance accounted for, rather than the reliable NSE variance: 0.2% instead of 0.3% and 2.0% instead of 4.7%. Genetics accounted for much more of the total variance: 5.0% for self‐rated symptoms and 11.0% for parent‐rated symptoms.

We found similar patterns of results for predictions from preschool to childhood and from childhood to adolescence for NSE, genetic and shared environmental processes. On average, parent‐rated poly‐E measures accounted for 1.5% of the reliable NSE variance in parent ratings of symptoms of behaviour problems in childhood, 0.2% in teacher ratings and 0.3% in self‐ratings, after controlling for genetics, shared environment and error of measurement. In adolescence, the NSE predictions were 6.8% for parent‐rated, 0.8% for teacher‐rated and 1.8% for self‐rated behaviour problem symptoms. Results for adolescence‐to‐adulthood analyses were consistently weaker, but this is most likely due to our weaker assessment of the environment in adolescence.

For the specific measures used in our study, we conclude that preschool, primary and secondary school environments do not have a major environmental impact, whether NSE or shared environment, on behaviour problem outcomes in adulthood. The strongest predictive processes are genetic. Similar results have been found in previous research, for example, predicting depressive symptoms (Pike, McGuire, et al., 1996), externalising disorders (Burt et al., 2003) and antisocial behaviour (Narusyte et al., 2007; Pike, McGuire, et al., 1996).

These results are limited to the normal range of environmental variation and cannot be assumed to generalise to environmental extremes of neglect, abuse or catastrophic events. Some research supports the possibility that NSE effects are greater in higher risk environments (Asbury, Dunn, Pike, & Plomin, 2003). Another limitation is that the measures of behaviour problems used in the present study, although standard measures often used in other research, are limited to questionnaire ratings by parents, teachers and the twins. Moreover, our measures of the environment are limited to ratings by parents. There is some evidence that observational measures yield stronger NSE results than questionnaires (Pike, McGuire, et al., 1996; Turkheimer & Waldron, 2000). On the other hand, self‐report questionnaires tap into perceptions, which is how the environment is experienced (Plomin, 1994) and aggregate information over time, as opposed to a few observed instances.

A general limitation for research on NSE is that measures of the family environment have traditionally focused on between‐family rather than within‐family environments specific to each child (Asbury, Moran, & Plomin, 2017; Daniels & Plomin, 1985). More measures of the within‐family environment are needed that are specific to each child in a family because there is no necessary relationship between the environmental causes of differences between families and the environmental causes of differences within families (Plomin & Daniels, 1987). One example of the within‐family NSE factor includes unequal distribution of affection from parents, measured based on siblings' perceptions (Plomin & Daniels, 1987).

At the least, our results can be seen as a challenge to researchers to account for more of the NSE variance in behaviour problem symptoms after controlling for genetics. This is an important goal because NSE is the way the environment works to affect symptoms of behaviour problems, not just for siblings but for all children. These results underline the need to control for the effects of genes because correlations between environmental measures and symptoms of behaviour problems are substantially (about 50%) mediated by genetic factors. More generally, these findings remind us that correlations between environmental measures and behaviour problem symptoms cannot be assumed to be environmentally causal.

The major question raised by this research is how we can narrow the large ‘missing NSE' gap between variance in behaviour problem symptoms explained by measured NSE and the NSE component of variance, especially if specific NSE factors, as we currently measure them, have miniscule effect sizes. One possibility has been called *the gloomy prospect*: ‘that the salient environment might be unsystematic, idiosyncratic or serendipitous events such as accidents, illnesses and other traumas’ (Plomin & Daniels, 1987, p. 8), which could include ‘intrinsic stochasticity of molecular processes’ (Tikhodeyev & Shcherbakova, 2019). We should not accept this null hypothesis of the gloomy prospect until we have exhausted attempts to prove it wrong, because NSE effects are real and the ‘missing NSE' gap might reflect our current inability to measure and detect systematic effects.

An instructive comparison is the ‘missing heritability’ gap (Manolio et al., 2009; Turkheimer, 2012), which refers to the disparity between variance in behaviour problem symptoms explained by measured DNA variants (about 4%) and their heritability (about 40%; Cheesman et al., 2017; Gidziela et al., 2022). The first wave of DNA research investigated candidate genes, which were assumed to have large effects, but this candidate gene research failed to yield replicable associations (Duncan & Keller, 2011). Most NSE research is at an analogous ‘candidate NSE' stage, testing for large effects of the usual suspects such as parenting and peers.

One possibility to narrow the ‘missing heritability’ gap came with a technological advance, the DNA chip, which enabled the systematic strategy of genome‐wide association (GWA) studies (Plomin, 2019). GWA analyses revealed that the largest associations were much smaller than anyone imagined (Visscher et al., 2017). A technological advance comparable to the DNA chip that could create a similar breakthrough for NSE research is the RNA chip, which makes it possible to adopt a systematic approach analogous to the DNA chip and GWA analysis by assessing the expression levels of all 30,000 genes in the genome (von Stumm & d'Apice, 2022). Crucially, gene expression is responsive to the endogenous and exogenous environment (Feil & Fraga, 2012). In this way, RNA chips can provide a genome‐wide snapshot of environmental effects. However, gene expression reflects a momentary state because RNA transcripts degrade quickly, the better to reflect changes in the environment. A more focused starting point is the slow‐motion gene expression changes involving epigenetic mechanisms, which can be assessed via DNA methylation marks and which are substantially due to NSE (Bell & Spector, 2011; Wong et al., 2014). A major limitation is that both transcriptomics and epigenomics are tissue specific, and the tissue that most interests psychologists is the brain, which is not accessible except post mortem.

Another solution to the ‘missing NSE' gap could come from technological advances in remote real‐time biological and behavioural monitoring using wearable devices and smartphones and in digital footprints left in social media (Adjerid & Kelley, 2018). New analytic approaches such as machine learning can make sense of these massive datasets, especially in relation to prediction rather than explanation (Yarkoni & Westfall, 2017).

A limitation of any attempt to identify NSE causes of behaviour problem symptoms is that it is difficult to establish causality (Turkheimer & Waldron, 2000). For this reason, we have refrained from interpreting NSE‐mediated correlations between environmental measures and behaviour problem symptoms as causal, even though we correlated environmental measures at one age with behaviour problem symptoms at a later age. Our goal is to identify NSE factors that predict symptoms of behaviour problems, which is a prerequisite for explaining these associations. Moreover, in our view, prediction is a more tractable and practical goal than explanation for understanding the major source of variance in symptoms of children's behaviour problems – non‐shared environment.

## Supporting information


**Appendix S1.** Statement of hypotheses preregistered with the Open Science Framework.
**Appendix S2.** Description of the TEDS sample.
**Appendix S3.** Selection of environmental measures.
**Appendix S4.** Construction of the poly‐E composites.
**Appendix S5.** Description of univariate and multivariate twin analyses.
**Appendix S6.** Description of MZ differences analyses.
**Appendix S7.** Results of MZ differences analyses.
**Figure S1.** Distribution of correlations between envirornmental measures and symptoms of behaviour problems.
**Figure S2.** Behaviour problem measures and their composites across ages.
**Figure S3.** Cholesky decomposition models.
**Figure S4.** Path diagrams of the bivariate Cholesky model.
**Figure S5.** Phenotypic correlations between poly‐E composites (i.e., environmental measures) and behaviour problem symptoms.
**Figure S6.** Path diagrams of the multivariate Cholesky model.
**Figure S7.** Correlations between MZ difference scores.
**Figure S8.** Comparison of results obtained from MZ differences, residualised scores and Cholesky analyses.
**Figure S9.** Correlations between residual MZ scores.
**Table S1.** Representativeness of the selected sample used in the present study.
**Table S2.** Environmental measures selected to create poly‐E composites specific to each behaviour problem measure.
**Table S3.** Genetic, shared and nonshared environmental influences on behaviour problem symptoms estimated for the total sample.
**Table S4.** Genetic, shared and nonshared environmental influences on behaviour problem symptoms estimated for males.
**Table S5.** Genetic, shared and nonshared environmental influences on behaviour problem symptoms estimated for females.
**Table S6.** Genetic, shared and nonshared environmental squared bivariate path estimates calculated for the total sample.
**Table S7.** Genetic, shared and nonshared environmental squared bivariate path estimates calculated for males.
**Table S8.** Genetic, shared and nonshared environmental squared bivariate path estimates calculated for females.
**Table S9.** Genetic, shared and nonshared environmental standardised squared multivariate path estimates for the total sample for the cumulative NSE prediction of behaviour problem symptoms in adulthood from environmental measures in preschool, childhood and adolescence.
