# Supplementary material for: Explaining the influence of non‐shared environment (NSE) on symptoms of behaviour problems from preschool to adulthood: mind the missing NSE gap
Source: J Child Psychol Psychiatry. 2022 Nov 27;64(5):747–57. doi: 10.1111/jcpp.13729 (PMC10953036; doi:10.1111/jcpp.13729)
Supplement: Supplementary file 1 [file JCPP-64-747-s001.docx]

**Explaining the influence of nonshared environment (NSE) on symptoms of behaviour problems from preschool to adulthood: Mind the missing NSE gap.**

Agnieszka Gidziela^1,2^, Margherita Malanchini^1,2^, Kaili Rimfeld^2,3^, Andrew McMillan^2^, Angelica Ronald^4^, Essi Viding^5^, Alison Pike^6^, Kathryn Asbury^7^, Thalia C. Eley^2^, Sophie von Stumm^7^ and Robert Plomin^2^

^1 ^School of Biological and Chemical Sciences, Queen Mary University of London

^2 ^Social, Genetic & Developmental Psychiatry Centre, Institute of Psychiatry, Psychology 

& Neuroscience, King’s College London 

^3^ Department of Psychology, Royal Holloway University of London

^4 ^Department of Psychological Sciences, Birkbeck University of London

^5 ^Division of Psychology and Language Sciences, University College London

^6 ^School of Psychology, University of Sussex

^7 ^Department of Education, University of York

**Supporting information** 

**Appendix S1.** Statement of hypotheses pre-registered with the Open Science Framework.	3

**Appendix S2.** Description of the TEDS sample.	6

**Appendix S3.** Selection of environmental measures.	7

**Appendix S4.** Construction of the poly-E composites.	9

**Appendix S5.** Description of univariate and multivariate twin analyses.	10

**Appendix S6.** Description of MZ differences analyses.	12

**Appendix S7.** Results of MZ differences analyses.	13

**Appendix S****1****.** Statement of hypotheses pre-registered with the Open Science Framework.

The following hypotheses were pre-registered with the Open Science Framework (OSF; [https://osf.io/rbv9q](https://osf.io/rbv9q)). Here, we address each hypothesis according to obtained results.

Hypothesis 1*: In univariate twin analyses of behaviour problems, nonshared environment (NSE) accounts for more variance in behaviour problems than nurture (shared environment) and nature (genetics) combined.*

Hypothesis 1 was only supported for self ratings of behaviour problem symptoms across ages (average NSE influence of 59%), but not parent or teacher ratings (37% and 40%, respectively)

Hypothesis 2: *In bivariate twin analyses between environmental measures and behaviour problems, NSE-mediated links cumulatively account for significant but modest (<5%) total variance in behaviour problems.*

Hypothesis 2 was supported. On average across childhood, adolescence and adulthood, NSE-mediated links accounted for 1% of the total variance in parent-rated behaviour problem symptoms, 0.2% in teacher-rated behaviour problem symptoms and 0.5% in self-rated behaviour problem symptoms. 

Hypothesis 3: *In bivariate twin analyses between environmental measures and behaviour problems, links mediated by shared environment cumulatively account for significant but modest (<5%) total variance in behaviour problems.* 

Hypothesis 3 was supported. On average across childhood, adolescence and adulthood, links mediated by shared environment accounted for 2% of the total variance in parent-rated behaviour problem symptoms, 0.4% in teacher-rated behaviour problem symptoms and 0.9% in self-rated behaviour problem symptoms. 

Hypothesis 4: *Most of the association between environmental measures and behaviour problems is mediated genetically.*  

Hypothesis 4 was supported. On average across childhood, adolescence and adulthood, links mediated by shared environment accounted for 11.9% of the total variance in parent-rated behaviour problem symptoms, 5.1% in teacher-rated behaviour problem symptoms and 5.3% in self-rated behaviour problem symptoms. 

Hypothesis 5: *The MZ differences design yields comparable results about the E-mediated links between environmental measures and behavioural problems, although the MZ differences design does not assess the extent to which the total variance of behavioural problems is explained by E-related processes.*

Hypothesis 5 was supported. On average, poly-E MZ difference scores predicted 3.2% of the variance in parent-rated, 0.4% in teacher-rated and 0.9% In self-rated MZ differences in symptoms of childhood behaviour problems. Our Cholesky analyses estimated that on average across childhood, adolescence and adulthood, parent-rated NSE measures accounted for 3.4% of the reliable NSE variance in parent-rated behaviour problem symptoms, 0.3% in teacher-rated behaviour problem symptoms and 0.9% in self-rated behaviour problem symptoms.

For each of these hypotheses, we predict:

a) Results are stronger when the same person (parent, teacher, child) rates 

the environment and behaviour problems as compared to cross-rater analyses.

b) Results are similar for males and females. 

We have no hypotheses about:

a) Differential results for specific behaviour problems (emotional problemshyperactivity/attention problems, conduct problems and peer problems).

b) Developmental trends across the three ages.

**Appendix S****2****.** Description of the TEDS sample.

Our sampling frame consisted of up to 4039 pairs of twins born in England and Wales between 1994 and 1996 who have been enrolled in the Twins Early Development Study (TEDS) (Rimfeld et al., 2019).

The TEDS twins have been assessed a dozen times from infancy through early adulthood on a wide range of behavioural, psychological, cognitive, physical and environmental measures (Rimfeld et al., 2019). Data collection procedures included questionnaires administered by post, by telephone and online, as described in an overview of TEDS (Rimfeld et al., 2019). Details can be found in the TEDS data dictionary: [https://www.teds.ac.uk/datadictionary/home.htm](https://www.teds.ac.uk/datadictionary/home.htm)). 

The sample of TEDS twins is representative of the UK population in terms of ethnicity and socioeconomic status (SES) (**Table S1)**; for details of representativeness and attrition, see Rimfeld et al. (2019). Individuals with severe medical conditions were excluded from analyses. These conditions include detrimental prenatal and postnatal conditions, as well as other conditions that could seriously impact later development. In addition, twins with uncertain and unknown zygosity were excluded from the analyses. Zygosity was recorded using a parent questionnaire of physical similarity between twins, with 95% accuracy when ascertained by DNA tests (Price et al., 2000). 

**Appendix S****3****.** Selection of environmental measures.

In order to create composite environmental measures in preschool, childhood and adolescence, we reduced the number of  environmental items from several hundred to fewer than one hundred using two criteria. 

Our first criterion was a moderate phenotypic correlation between environmental measures and behaviour problem symptoms. We correlated environmental measures in preschool with symptoms of each of the four behaviour problems in childhood, environmental measures in childhood with behaviour problem symptoms in adolescence and environmental measures in adolescence with behaviour problem symptoms in adulthood for each rater (parent, teacher, and self report) separately. We explored the distributions of correlation coefficients (**Figure S1**) and used r= 0.20 as a cut-off above which environmental measures were included. 

The second criterion excluded highly correlated environmental measures. We used penalized elastic net regularization with training and test iterations (see Zou & Hastie, 2005; Allegrini et al., 2020 and Gidziela et al., 2021) to predict symptoms of each of the four behaviour problems in childhood from environmental measures in preschool, behaviour problem symptoms in adolescence from environmental measures in childhood and behaviour problem symptoms in adulthood from environmental measures in adolescence. Elastic net regularization accounts for intercorrelations between environmental predictors (multicollinearity) by deleting redundant measures. 

**Table S2** describes the environmental variables selected to be included in the poly-environmental (poly-E) composite for each of the four target behaviour problem symptoms at each age**.** Additional information about environmental measures can be found in the TEDS data dictionary (https://www.teds.ac.uk/datadictionary/home.htm).

**Appendix S****4****.** Construction of the poly-E composites.

We used a penalized elastic net regularization to predict parent-rated behaviour problem symptoms in childhood from parent-rated E measures in preschool, parent-rated behaviour problem symptoms in adolescence from parent-rated E measures in childhood and parent-rated behaviour problem symptoms in adulthood from parent-rated E measures in adolescence. Elastic net regularization accounts for intercorrelations between predictors (multicollinearity) by deleting redundant measures.  

We used regression weights to create poly-E composites in preschool, childhood and adolescence: 

${poly-E}_{i}= \sum_{j=1}^{k}{E_{ij}β_{j}}$

where $poly-E$ is the poly-E measure for individual $i$ in the full sample, $j=$ {1, 2, …} and denotes the E value for the k E measures for individual $i$ and $ß$ indicates the elastic net coefficient of the association between the $jth$ predictor E and the behaviour problem measure.

**Appendix S****5****.** Description of univariate and multivariate twin analyses.

*The univariate twin method to estimate ‘anonymous’ (unmeasured) NSE influences*

The twin method allows for the decomposition of individual differences in a trait into genetic and environmental sources of variance by capitalizing on the genetic relatedness between monozygotic twins (MZ), who share 100% of their genetic makeup, and dizygotic twins (DZ), who share on average 50% of the genes that differ between individuals (Knopik et al., 2017). By comparing how similar MZ and DZ twins are for a given trait, it is possible to estimate the relative contribution of genetic factors and environments to variation in that trait. Heritability, the amount of variance in a trait that can be attributed to genetic variance (A), can be roughly estimated as double the difference between the MZ and DZ twin correlations. The variance can be further partitioned into shared environment (C), which describes the extent to which twins raised in the same family resemble each other beyond their shared genetic variance, and non-shared environment (E), which describes environmental variance that does not contribute to similarities between twin pairs (and also includes measurement error). The twin model-fitting analyses were conducted using OpenMx for R (Neale et al., 2016; R Core Team, 2021).

*The multivariate twin method to estimate the NSE-related covariance between measures of environment and behaviour problem symptoms after controlling for genetics and shared environment*

Multivariate (Cholesky) twin model-fitting is theoretically akin to hierarchical regression (Rijsdijk & Sham, 2002) in the sense that the degree to which NSE influences on a measure of behaviour problem symptoms can be explained by the NSE influences on a measure of the environment. The bivariate model as illustrated in **Figure S3A**, called a Cholesky model, decomposes the genetic and environmental variance in behaviour problem symptoms) into two components of variance: one component that is shared with the environmental measure and a component that is independent of the environmental measure. The sum of squared paths (standardised partial regressions) *e*_*12*_ and *e*_*22*_ estimates the NSE variance in the measure of behaviour problems, equivalent to NSE influence estimated from the univariate twin model. Squaring the path *e*_*12*_ estimates NSE-related processes in behaviour problem symptoms attributable to the environmental measure. Dividing this estimate (i.e., squared path *e*_*12*_) by the NSE estimate of behaviour problem symptoms (i.e., the sum of squared paths *e*_*12*_ and *e*_*22*_) results in the proportion of NSE variance accounted for by the environmental measure. Squaring the path from the residual latent variable *e*_*22*_ estimates NSE-related processes in the measure of behaviour problem symptoms independent of the environmental variable, which includes error of measurement. 

By simultaneously including poly-E measures in preschool, childhood and adolescence as predictors of a measure of symptoms of behaviour problems in adulthood, we can estimate cumulative NSE influence on the measure of behaviour problem symptoms **(Figure S3B**).

**Appendix S****6****.** Description of MZ differences analyses.

The MZ differences design correlates MZ differences on an environmental measure with MZ differences on a measure of symptoms of behaviour problems. It provides a within-family estimate of the E latent variable in **Figure S3A** because MZ twins reared together differ due to NSE because they are identical in terms of inherited DNA differences and shared environmental influences are those that make members of an MZ twin pair similar, not different (Vitaro et al., 2009).  

To assess the NSE influence on a trait using the MZ differences design, MZ co-twins were randomly assigned as Twin 1 or Twin 2, followed by calculation of a relative difference scored by subtracting Twin 2’s score from Twin 1’s score for both the environmental measure and the measure of behaviour problems. These two difference scores were then correlated to estimate NSE-mediated links between the environmental measure and the measure of behaviour problems. Although previously criticized for reliability concerns (Bereiter, 1963), the MZ differences design is thought to provide a reliable and unbiased approximation of differences (Rovine, 1994). A variant of the MZ differences design regresses Twin 1’s scores on Twin 2’s scores, resulting in standardized residuals as an index of within-pair discrepancy. Residuals obtained for the environmental measure and the measure of behavioural problems are then correlated to estimate NSE-mediated links. This alternative design is expected to yield results similar to the simple MZ differences design (Turkheimer & Waldron, 2000).

**Appendix S****7****.** Results of MZ differences analyses.

On average, preschool poly-E MZ difference scores predicted 1.5% of the variance in parent-rated, 0.2% in teacher-rated and 0.4% in self-rated MZ differences in childhood behaviour problem symptoms. Our Cholesky analyses estimated on average that preschool poly-E scores predicted equivalent amount of variance. Similarly, for childhood poly-E and symptoms of adolescent behaviour problems, on average MZ differences in poly-E scores predicted 6.1% of the variance of MZ differences for parent-rated behaviour problems, 0.7% for teacher-rated behaviour problems and 2% in self-rated behaviour problems. Our corresponding Cholesky results were 6.8%, 0.8% and 1.8%, respectively. Finally, for adolescent poly-E and adult behaviour problem symptoms, on average, parent-rated poly-E MZ difference scores predicted 0.6% of the variance in parent-rated and 0.1% in self-rated behaviour problems MZ difference scores. These are the same as the results from our Cholesky analyses. As illustrated in **Figure S8**, similar results were obtained from residualised MZ scores and MZ differences analyses.

**Supporting figures**

**Figure S1.** Distribution of correlations between envirornmental measures and symptoms of behaviour problems.	14

**Figure S2.** Behaviour problem measures and their composites across ages.	15

**Figure S3.** Cholesky decomposition models.	16

**Figure S4.** Path diagrams of the bivariate Cholesky model.	17

**Figure S5.** Phenotypic correlations between poly-E composites (i.e., environmental measures) and behaviour problem symptoms.	18

**Figure S6.** Path diagrams of the multivariate Cholesky model.	19

**Figure S7.** Correlations between MZ difference scores.	20

**Figure S8.** Comparison of results obtained from MZ differences, residualised scores and Cholesky analyses.	22

**Figure S9.** Correlations between residual MZ scores.	23

**Figure S****1****.** Distribution of correlations between envirornmental measures and symptoms of behaviour problems.

Histograms illustrating distributions of correlations between parent-rated environmental measures in preschool, childhood and adolescence and parent-rated behaviour problem symptoms measured at the subsequent age (e.g.environmental measures in preschool predicting behaviour problem symptoms in childhood). Numbers on top of the bars represent the number of environmental variables reaching the correlation with symptoms of behaviour problems as indicated on the x-axis.

|  |
| --- |

**Figure S****2****.** Behaviour problem measures and their composites across ages.

|  |
| --- |

**Figure S****3****.** Cholesky decomposition models.

Panel **A** presents the bivariate genetic model (Cholesky decomposition) of a measure of environment and a measure of behaviour problem symptoms. A_1_, C_1_ and E_1_ are latent factors indexing genetic (A), common or shared environmental (C) and nonshared environmental (E) variance for the environmental measure . A_2_, C_2_ and E_2_ are latent factors indexing residual variance in the measure of behaviour problems independent of the environmental measure.

Panel **B** presents a multivariate Cholesky model of poly-E composites (i.e., environmental measures) in preschool, childhood and adolescence cumulatively predicting a measure of behaviour problem symptoms in adulthood. E_1_ is a latent factor indexing nonshared environmental factors influencing all four measures, while E_4_ is a latent factor indexing nonshared environmental factors specific to the measure of behaviour problems in adulthood after accounting for poly-E composites. For simplicity, this figure illustrates only the NSE components of variance and covariance.

|  |
| --- |
|  |

**Figure S****4****.** Path diagrams of the bivariate Cholesky model.

Bivariate Cholesky model of poly-E composites (i.e., environmental measures) in preschool (**A**), childhood (**B**) and adolescence (**C**) and measures of behaviour problems in subsequent developmental stages. For simplicity, only the NSE components of variance and covariance are illustrated. Presented path estimates are standardised. Estimates in this figure are square roots of the path estimates in **Table S6**.

|  |
| --- |

**Figure S****5****.** Phenotypic correlations between poly-E composites (i.e., environmental measures) and behaviour problem symptoms.

Correlations between poly-E composites (constructed for hyperactivity, conduct, emotional problems and peer problems) and behaviour problem symptoms (i.e., correlations between poly-E composites in preschool, childhood and adolescence and hyperactivity, conduct, emotional problems and peer problems at the same age, as well as subsequent ages).

|  |
| --- |

**Figure S****6****.** Path diagrams of the multivariate Cholesky model.

Multivariate Cholesky analysis of poly-E composites (i.e., environmental measures) in preschool, childhood and adolescence cumulatively predicting hyperactivity and conduct in adulthood. E_1_ is a latent factor indexing nonshared environmental factors influencing all traits while E_4_ is a latent factor indexing nonshared environmental factors specific to behaviour problems measure. For simplicity, only the NSE components of variance and covariance are illustrated. Presented path estimates are standardised. Estimates in this figure are square roots of the path estimates in **Table S9.**

|  |
| --- |

**Figure S****7****.** Correlations between MZ difference scores.

Correlations between MZ poly-E differences (i.e., environmental measures constructed for hyperactivity, conduct, emotional problems and peer problems) and MZ behaviour problem symptom differences in childhood (**A**), adolescence (**B**) and adulthood (**C**).

|  |
| --- |
|  |
|  |

**Figure S****8****.** Comparison of results obtained from MZ differences, residualised scores and Cholesky analyses.

| ***Note.*** Variance explained in behaviour problems refers to the proportion of NSE variance explained by poly-E composites, not the total variance. For a detailed description, see **Appendix S5.** |
| --- |
|  |

**Figure S****9****.** Correlations between residual MZ scores.

Correlations between residual MZ poly-E scores (i.e., environmental measures constructed for hyperactivity, conduct, emotional problems and peer problems) and residual MZ behaviour problem symptom scores in childhood (**A**), adolescence (**B**) and adulthood (**C**).

|  |
| --- |
|  |
|  |

**Supporting tables**

**Table S1.** Representativeness of the selected sample used in the present study.	26

**Table S2.** Environmental measures selected to create poly-E composites specific to each behaviour problem measure.	27

**Table S3.** Genetic, shared and nonshared environmental influences on behaviour problem symptoms estimated for the total sample.	31

**Table S4.** Genetic, shared and nonshared environmental influences on behaviour problem symptoms estimated for males.	33

**Table S5.** Genetic, shared and nonshared environmental influences on behaviour problem symptoms estimated for females.	35

**Table S6.** Genetic, shared and nonshared environmental squared bivariate path estimates calculated for the total sample.	37

**Table S7.** Genetic, shared and nonshared environmental squared bivariate path estimates calculated for males.	41

**Table S8.** Genetic, shared and nonshared environmental squared bivariate path estimates calculated for females.	45

**Table S9.** Genetic, shared and nonshared environmental standardised squared multivariate path estimates for the total sample for the cumulative NSE prediction of behaviour problem symptoms in adulthood from environmental measures in preschool, childhood and adolescence.	49

**Table S****1****.** Representativeness of the selected sample used in the present study.

| Ethnicity and SES | **Selected sample** | **1st Contact sample** | **National equivalents**^**ab**^ |
| --- | --- | --- | --- |
| % white | 94.9% | 91.7% | 93% |
| % mother A-levels or higher | 50.4% | 35.5% | 35% |
| % father A-levels or higher | 54.8% | 44.8% | 47% |
| % mother employed | 50.2% | 43.1% | 50% |
| % father employed | 94.9% | 91.6% | 91% |
| Note. ^a^ including cohort of parents with children born in late 1990s and early 2000s; ^b^  from Rimfeld et al. (2019). The selected sample is the sample used in the present analyses; The first contact sample is the sample who responded to the first TEDS contact when the twins were infants. |  |  |  |

**Table S****2****.** Environmental measures selected to create poly-E composites specific to each behaviour problem measure.

| **Target behaviour problem measure in childhood** | **Selected parent-rated environmental measures in preschool** |
| --- | --- |
|  |  |
| **Hyperactivity** | **Year 3:** Parental discipline (Deater–Deckard et al., 1998): smack/shout; Twin environment risk^1^ (Cox et al., 1987; Matheny Jr et al., 1995) **Year 4:** Parental discipline (Deater–Deckard et al., 1998): smack/shout; Twin environment risk^1^ (Cox et al., 1987; Matheny Jr et al., 1995) |
|  |  |
| **Conduct** | **Year 3:** Parental discipline (Deater–Deckard et al., 1998): smack/slap; Parental discipline overall scale (Deater–Deckard et al., 1998); Parental feelings overall scale (Deater–Deckard et al., 1998); Twin environment risk^1^ (Cox et al., 1987; Matheny Jr et al., 1995) **Year 4:** Parental discipline (Deater–Deckard et al., 1998): smack/shout; Parental discipline (Deater–Deckard et al., 1998): smack/slap; Parental discipline (Deater–Deckard et al., 1998): shout/tell off; Parental discipline overall scale (Deater–Deckard et al., 1998); Parental feelings (Deater–Deckard et al., 1998): angry; Parental negativity scale^2^ (Deater–Deckard et al., 1998); Parental feelings overall scale (Deater–Deckard et al., 1998); Twin environment risk^1^ (Cox et al., 1987; Matheny Jr et al., 1995) |
|  |  |
| **Emotional problems** | **Year 3:** Twin environment risk^1^ (Cox et al., 1987; Matheny Jr et al., 1995) **Year 4:** Twin environment risk^1^ (Cox et al., 1987; Matheny Jr et al., 1995) |
|  |  |
| **Peer problems** | **Year 3:** Twin environment risk^1^ (Cox et al., 1987; Matheny Jr et al., 1995) **Year 4:** Twin environment risk^1^ (Cox et al., 1987; Matheny Jr et al., 1995) |
| **Target behaviour problem measure in adolescence** | **Selected parent-rated environmental measures in childhood** |
|  |  |
| **Hyperactivity** | **Year 7:** Parental harsh discipline scale^3^ (Deater–Deckard et al., 1998); Parental negativity scale^2^ (Deater–Deckard et al., 1998); Parental feelings (Deater–Deckard et al., 1998) **Year 9:** Parental feelings (Deater–Deckard et al., 1998): feel impatient with child; Parental feelings (Deater–Deckard et al., 1998): wish child would leave alone; Parental feelings (Deater–Deckard et al., 1998): feel frustrated by child; Parental feelings overall scale (Deater–Deckard et al., 1998); Home environment: parent help with homework when needed; Home environment: homework should be done alone; Classroom environment (Ainley & Bourke, 1992): likes to be in a classroom; Classroom environment (Ainley & Bourke, 1992): feels happy in a classroom; Classroom environment (Ainley & Bourke, 1992): friends often in trouble; Classroom friends/peer context^4^ (Ainley & Bourke, 1992); Classroom adventure context^5^ (Ainley & Bourke, 1992); Classroom acceptance^6^ (Ainley & Bourke, 1992) |
|  |  |
| **Conduct** | **Year 7:** Parental discipline (Deater–Deckard et al., 1998): smack or restrain; Parental discipline (Deater–Deckard et al., 1998): send to room; Parental feelings scale (Deater–Deckard et al., 1998): angry; Parental harsh discipline scale^3^ (Deater–Deckard et al., 1998); Parental feelings overall scale (Deater–Deckard et al., 1998) **Year 9:** Parental feelings scale (Deater–Deckard et al., 1998): wish child would leave alone; Parental feelings scale (Deater–Deckard et al., 1998): child makes me angry; Parental feelings scale (Deater–Deckard et al., 1998): feel frustrated by child; Parental feelings overall scale (Deater–Deckard et al., 1998) |
|  |  |
| **Emotional problems** | **Year 9:** Classroom environment (Ainley & Bourke, 1992): feels worried; Classroom negative affect^7^ (Ainley & Bourke, 1992) |
|  |  |
| **Peer problems** | **Year 9:** Classroom environment (Ainley & Bourke, 1992): accepted by other children; Classroom environment (Ainley & Bourke, 1992): gets on well with children; Classroom environment (Ainley & Bourke, 1992): feels lonely; Classroom environment (Ainley & Bourke, 1992): feels happy; Classroom environment (Ainley & Bourke, 1992): people think a lot of; Classroom environment (Ainley & Bourke, 1992): popular with other children; Classroom negative affect^7^ (Ainley & Bourke, 1992); Classroom acceptance^6^ (Ainley & Bourke, 1992) |
|  |  |
| **Target behaviour problem measure in adulthood** | **Selected parent-rated environmental measures in adolescence** |
|  |  |
| **Hyperactivity** | **Year 12**: Parental feelings scale (Deater-Deckard, 2000): feel frustrated by child |
|  |  |
| **Conduct** | **Year 12**: Parental feelings scale (Deater-Deckard, 2000): child makes me angry; Parental feelings scale (Deater-Deckard, 2000): feel frustrated by child |
| *Note.* ^*1*^ Twin environment risk measure was computed as a standardized mean of family socio-economic status (SES), prenatal and perinatal medical risk, household chaos (the Confusion, Hubbub and Order Scale; Matheny Jr et al., 1995), maternal postnatal depression (the Edinburgh Postnatal Depression Scale; Cox et al., 1987), life events (such as changes to marital status, new siblings, mother's pregnancy, job changes and serious illness/accident), Parental feelings overall scale (Deater–Deckard et al., 1998) and Parental discipline overall scale (Deater–Deckard et al., 1998). ^2 ^Parental negativity scale was computed as a standardized mean of the following items from the Parental feelings overall scale (Deater–Deckard et al., 1998): angry, frustrated, impatient and wish the child would go away for a few minutes. ^3^ Parental harsh discipline scale was computed as a standardized mean of the following items from the Parental discipline overall scale (Deater–Deckard et al., 1998): smack or restrain, send the child to their room, shout or raise voice and ignore the child. ^4 ^Classroom friends/peer context scale was computed as a standardized mean of the following items from the Classroom Environment scale (Ainley & Bourke, 1992): child’s friends care about work, child’s friends try their best, child’s friends enjoy learning, child’s friends often in trouble. ^5 ^Classroom adventure context scale was computed as a standardized mean of the following items from the Classroom Environment scale (Ainley & Bourke, 1992): work is interesting, child gets excited about work, child likes to do extra work, child finds learning fun. ^6 ^Classroom acceptance scale was computed as a standardized mean of the following items from the Classroom Environment scale (Ainley & Bourke, 1992): child is accepted by other children, child gets on well with children, people think a lot of the child, child is popular with other children. ^6 ^Classroom negative affect scale was computed as a standardized mean of the following items from the Classroom Environment scale (Ainley & Bourke, 1992): child feels upset, child feels unhappy, child feels lonely, child feels worried. |  |

**Table S****3****.** Genetic, shared and nonshared environmental influences on behaviour problem symptoms and poly-E composites (i.e., environmental measures) estimated for the total sample.

| **Behaviour problem symptoms** | **h2 (95% CIs)** | **c2 (95% CIs)** | **e2 (95% CIs)** | **N total** | **N MZ** | **N DZ** |
| --- | --- | --- | --- | --- | --- | --- |
|  |  |  |  |  |  |  |
| Preschool hyperactivity parent-rated | 0.45 (0.42, 0.48) | 0.00 (0.00, 0.00) | 0.55 (0.52, 0.58) | 9004 | 3061 | 5943 |
| Preschool conduct problems parent-rated | 0.61 (0.56, 0.66) | 0.10 (0.05, 0.14) | 0.30 (0.28, 0.31) | 9000 | 3061 | 5939 |
| Preschool emotional problems parent-rated | 0.59 (0.54, 0.61) | 0.00 (0.00, 0.04) | 0.41 (0.39, 0.43) | 8999 | 3059 | 5940 |
| Preschool peer problems parent-rated | 0.59 (0.54, 0.61) | 0.00 (0.00, 0.04) | 0.41 (0.39, 0.43) | 8999 | 3059 | 5940 |
|  |  |  |  |  |  |  |
| Childhood hyperactivity parent-rated | 0.53 (0.50, 0.56) | 0.00 (0.00, 0.00) | 0.47 (0.44, 0.50) | 8100 | 2872 | 5228 |
| Childhood hyperactivity teacher-rated | 0.70 (0.68, 0.72) | 0.00 (0.00, 0.01) | 0.30 (0.28, 0.32) | 6673 | 2387 | 4286 |
| Childhood hyperactivity child-rated | 0.38 (0.34, 0.43) | 0.00 (0.00, 0.02) | 0.62 (0.57, 0.66) | 3306 | 1220 | 2082 |
| Childhood conduct problems parent-rated | 0.63 (0.59, 0.68) | 0.14 (0.09, 0.18) | 0.23 (0.22, 0.24) | 8106 | 2874 | 5232 |
| Childhood conduct problems teacher-rated | 0.69 (0.68, 0.71) | 0.00 (0.00, 0.04) | 0.31 (0.29, 0.32) | 6677 | 2387 | 4290 |
| Childhood conduct problems child-rated | 0.45 (0.34, 0.52) | 0.03 (0.00, 0.11) | 0.52 (0.48, 0.57) | 3305 | 1218 | 2087 |
| Childhood emotional problems parent-rated | 0.48 (0.42, 0.53) | 0.16 (0.11, 0.21) | 0.36 (0.34, 0.38) | 8103 | 2874 | 5229 |
| Childhood emotional problems teacher-rated | 0.51 (0.49, 0.54) | 0.00 (0.00, 0.02) | 0.49 (0.46, 0.51) | 6655 | 2375 | 4280 |
| Childhood emotional problems child-rated | 0.39 (0.27, 0.47) | 0.04 (0.00, 0.13) | 0.57 (0.53, 0.61) | 3303 | 1217 | 2086 |
| Childhood peer problems parent-rated | 0.67 (0.65, 0.69) | 0.00 (0.00, 0.01) | 0.33 (0.31, 0.35) | 8103 | 2874 | 5229 |
| Childhood peer problems teacher-rated | 0.66 (0.64, 0.68) | 0.00 (0.00, 0.03) | 0.34 (0.32, 0.36) | 6661 | 2382 | 4279 |
| Childhood peer problems child-rated | 0.31 (0.20, 0.43) | 0.08 (0.00, 0.17) | 0.60 (0.56, 0.65) | 3275 | 1210 | 2065 |
|  |  |  |  |  |  |  |
| Adolescence hyperactivity parent-rated | 0.74 (0.72, 0.75) | 0.00 (0.00, 0.00) | 0.26 (0.25, 0.28) | 6924 | 2480 | 4444 |
| Adolescence hyperactivity teacher-rated | 0.63 (0.59, 0.65) | 0.00 (0.00, 0.03) | 0.37 (0.35, 0.40) | 4319 | 1565 | 2754 |
| Adolescence hyperactivity child-rated | 0.46 (0.43, 0.49) | 0.00 (0.00, 0.01) | 0.54 (0.51, 0.57) | 6872 | 2460 | 4412 |
| Adolescence conduct problems parent-rated | 0.71 (0.66, 0.76) | 0.07 (0.03, 0.12) | 0.21 (0.20, 0.23) | 6927 | 2480 | 4447 |
| Adolescence conduct problems teacher-rated | 0.62 (0.57, 0.65) | 0.00 (0.00, 0.04) | 0.38 (0.35, 0.41) | 4322 | 1566 | 2756 |
| Adolescence conduct problems child-rated | 0.47 (0.43, 0.49) | 0.00 (0.00, 0.03) | 0.53 (0.51, 0.56) | 6872 | 2460 | 4412 |
| Adolescence emotional problems parent-rated | 0.56 (0.48, 0.62) | 0.05 (0.00, 0.10) | 0.40 (0.37, 0.42) | 5826 | 2115 | 3711 |
| Adolescence emotional problems teacher-rated | 0.44 (0.39, 0.47) | 0.00 (0.00, 0.03) | 0.56 (0.53, 0.60) | 4312 | 1566 | 2746 |
| Adolescence emotional problems child-rated | 0.41 (0.34, 0.44) | 0.00 (0.00, 0.05) | 0.59 (0.56, 0.62) | 6873 | 2460 | 4413 |
| Adolescence peer problems parent-rated | 0.74 (0.72, 0.75) | 0.00 (0.00, 0.02) | 0.26 (0.25, 0.28) | 5826 | 2115 | 3711 |
| Adolescence peer problems teacher-rated | 0.56 (0.53, 0.59) | 0.00 (0.00, 0.02) | 0.44 (0.41, 0.47) | 4317 | 1565 | 2752 |
| Adolescence peer problems child-rated | 0.50 (0.45, 0.52) | 0.00 (0.00, 0.04) | 0.50 (0.48, 0.53) | 6872 | 2460 | 4412 |
|  |  |  |  |  |  |  |
| Adulthood hyperactivity parent-rated | 0.58 (0.55, 0.61) | 0.00 (0.00, 0.01) | 0.42 (0.39, 0.45) | 5295 | 1896 | 3399 |
| Adulthood hyperactivity child-rated | 0.34 (0.31, 0.38) | 0.00 (0.00, 0.07) | 0.66 (0.62, 0.69) | 3879 | 1501 | 2378 |
| Adulthood conduct problems parent-rated | 0.55 (0.48, 0.58) | 0.00 (0.00, 0.05) | 0.45 (0.42, 0.48) | 5303 | 1898 | 3405 |
| Adulthood conduct problems child-rated | 0.27 (0.16, 0.31) | 0.00 (0.00, 0.08) | 0.73 (0.69, 0.77) | 3881 | 1501 | 2380 |
| Adulthood emotional problems parent-rated | 0.53 (0.49, 0.55) | 0.00 (0.00, 0.02) | 0.47 (0.45, 0.50) | 5291 | 1891 | 3400 |
| Adulthood emotional problems child-rated | 0.31 (0.19, 0.38) | 0.04 (0.00, 0.12) | 0.66 (0.62, 0.70) | 3881 | 1501 | 2380 |
| Adulthood peer problems parent-rated | 0.68 (0.65, 0.70) | 0.00 (0.00, 0.02) | 0.32 (0.30, 0.35) | 5296 | 1896 | 3400 |
| Adulthood peer problems child-rated | 0.39 (0.29, 0.43) | 0.00 (0.00, 0.08) | 0.61 (0.57, 0.64) | 3881 | 1501 | 2380 |
|  |  |  |  |  |  |  |
| **Poly-E composites** | **h2 (95% CIs)** | **c2 (95% CIs)** | **e2 (95% CIs)** | **N total** | **N MZ** | **N DZ** |
|  |  |  |  |  |  |  |
| Preschool hyperactivity poly-E | 0.30 (0.27, 0.33) | 0.60 (0.58, 0.63) | 0.10 (0.09, 0.10) | 8100 | 2872 | 5228 |
| Preschool conduct problems poly-E | 0.37 (0.33, 0.41) | 0.50 (0.46, 0.53) | 0.13 (0.12, 0.14) | 8106 | 2874 | 5232 |
| Preschool emotional problems poly-E | 0.10 (0.09, 0.11) | 0.86 (0.85, 0.87) | 0.04 (0.04, 0.05) | 8103 | 2874 | 5229 |
| Preschool peer problems poly-E | 0.10 (0.09, 0.11) | 0.86 (0.85, 0.87) | 0.04 (0.04, 0.05) | 8103 | 2874 | 5229 |
|  |  |  |  |  |  |  |
| Childhood hyperactivity poly-E | 0.70 (0.64, 0.77) | 0.15 (0.08, 0.21) | 0.15 (0.13, 0.16) | 6924 | 2480 | 4444 |
| Childhood conduct problems poly-E | 0.65 (0.59, 0.72) | 0.20 (0.13, 0.26) | 0.15 (0.14, 0.17) | 6927 | 2480 | 4447 |
| Childhood emotional problems poly-E | 0.33 (0.26, 0.41) | 0.34 (0.28, 0.40) | 0.33 (0.30, 0.36) | 5826 | 2115 | 3711 |
| Childhood peer problems poly-E | 0.63 (0.55, 0.71) | 0.07 (0.00, 0.14) | 0.30 (0.27, 0.33) | 5826 | 2115 | 3711 |
|  |  |  |  |  |  |  |
| Adolescence hyperactivity poly-E | 0.46 (0.42, 0.50) | 0.40 (0.37, 0.44) | 0.14 (0.13, 0.15) | 5295 | 1896 | 3399 |
| Adolescence conduct problems poly-E | 0.43 (0.40, 0.47) | 0.43 (0.39, 0.46) | 0.14 (0.13, 0.15) | 5303 | 1898 | 3405 |
| *Note.* h2= genetic influences; c2= shared environmental influences; e2= nonshared environmental influences; CIs= confidence intervals; N= sample size; MZ= monozygotic twin pairs; DZ= dizygotic twin pairs. |  |  |  |  |  |  |

**Table S****4****.** Genetic, shared and nonshared environmental influences on behaviour problem symptoms and poly-E composites (i.e., environmental measures) estimated for males.

| **Behaviour problem symptoms** | **h2 (95% CIs)** | **c2 (95% CIs)** | **e2 (95% CIs)** | **N total** | **N MZ** | **N DZ** |
| --- | --- | --- | --- | --- | --- | --- |
|  |  |  |  |  |  |  |
| Preschool hyperactivity parent-rated | 0.55 (0.50, 0.59) | 0.00 (0.00, 0.01) | 0.45 (0.41, 0.50) | 2935 | 1419 | 1516 |
| Preschool conduct problems parent-rated | 0.72 (0.66, 0.75) | 0.00 (0.00, 0.06) | 0.28 (0.25, 0.30) | 2936 | 1419 | 1517 |
| Preschool emotional problems parent-rated | 0.59 (0.55, 0.62) | 0.00 (0.00, 0.02) | 0.41 (0.38, 0.45) | 2933 | 1417 | 1516 |
| Preschool peer problems parent-rated | 0.59 (0.55, 0.62) | 0.00 (0.00, 0.02) | 0.41 (0.38, 0.45) | 2933 | 1417 | 1516 |
|  |  |  |  |  |  |  |
| Childhood hyperactivity parent-rated | 0.59 (0.55, 0.62) | 0.00 (0.00, 0.01) | 0.41 (0.38, 0.45) | 2649 | 1351 | 1298 |
| Childhood hyperactivity teacher-rated | 0.72 (0.70, 0.75) | 0.00 (0.00, 0.04) | 0.28 (0.25, 0.30) | 2182 | 1118 | 1064 |
| Childhood hyperactivity child-rated | 0.41 (0.32, 0.48) | 0.00 (0.00, 0.06) | 0.59 (0.52, 0.66) | 1046 | 536 | 510 |
| Childhood conduct problems parent-rated | 0.63 (0.55, 0.72) | 0.15 (0.06, 0.23) | 0.22 (0.20, 0.24) | 2651 | 1351 | 1300 |
| Childhood conduct problems teacher-rated | 0.71 (0.61, 0.74) | 0.00 (0.00, 0.10) | 0.29 (0.26, 0.31) | 2182 | 1118 | 1064 |
| Childhood conduct problems child-rated | 0.34 (0.15, 0.52) | 0.13 (0.00, 0.29) | 0.53 (0.47, 0.59) | 1045 | 534 | 511 |
| Childhood emotional problems parent-rated | 0.52 (0.42, 0.62) | 0.12 (0.02, 0.20) | 0.36 (0.34, 0.39) | 2651 | 1351 | 1300 |
| Childhood emotional problems teacher-rated | 0.50 (0.46, 0.54) | 0.00 (0.00, 0.03) | 0.50 (0.46, 0.54) | 2174 | 1113 | 1061 |
| Childhood emotional problems child-rated | 0.08 (0.00, 0.29) | 0.26 (0.12, 0.37) | 0.66 (0.59, 0.73) | 1044 | 534 | 510 |
| Childhood peer problems parent-rated | 0.64 (0.61, 0.67) | 0.00 (0.00, 0.02) | 0.36 (0.33, 0.39) | 2651 | 1351 | 1300 |
| Childhood peer problems teacher-rated | 0.67 (0.59, 0.70) | 0.00 (0.00, 0.07) | 0.33 (0.30, 0.36) | 2174 | 1116 | 1058 |
| Childhood peer problems child-rated | 0.23 (0.02, 0.43) | 0.14 (0.00, 0.30) | 0.63 (0.56, 0.71) | 1031 | 526 | 505 |
|  |  |  |  |  |  |  |
| Adolescence hyperactivity parent-rated | 0.75 (0.72, 0.77) | 0.00 (0.00, 0.01) | 0.25 (0.23, 0.28) | 2156 | 1123 | 1033 |
| Adolescence hyperactivity teacher-rated | 0.64 (0.60, 0.68) | 0.00 (0.00, 0.06) | 0.36 (0.32, 0.40) | 1345 | 700 | 645 |
| Adolescence hyperactivity child-rated | 0.48 (0.42, 0.52) | 0.00 (0.00, 0.04) | 0.52 (0.48, 0.56) | 2136 | 1112 | 1024 |
| Adolescence conduct problems parent-rated | 0.77 (0.68, 0.79) | 0.00 (0.00, 0.09) | 0.23 (0.21, 0.25) | 2156 | 1123 | 1033 |
| Adolescence conduct problems teacher-rated | 0.66 (0.62, 0.69) | 0.00 (0.00, 0.10) | 0.34 (0.31, 0.38) | 1347 | 701 | 646 |
| Adolescence conduct problems child-rated | 0.48 (0.43, 0.52) | 0.00 (0.00, 0.07) | 0.52 (0.48, 0.57) | 2136 | 1112 | 1024 |
| Adolescence emotional problems parent-rated | 0.59 (0.48, 0.63) | 0.00 (0.00, 0.10) | 0.41 (0.37, 0.45) | 1837 | 956 | 881 |
| Adolescence emotional problems teacher-rated | 0.45 (0.34, 0.50) | 0.00 (0.00, 0.09) | 0.55 (0.50, 0.61) | 1341 | 701 | 640 |
| Adolescence emotional problems child-rated | 0.37 (0.24, 0.42) | 0.00 (0.00, 0.11) | 0.63 (0.58, 0.67) | 2136 | 1112 | 1024 |
| Adolescence peer problems parent-rated | 0.73 (0.68, 0.75) | 0.00 (0.00, 0.04) | 0.27 (0.25, 0.30) | 1838 | 956 | 882 |
| Adolescence peer problems teacher-rated | 0.58 (0.44, 0.62) | 0.00 (0.00, 0.12) | 0.42 (0.38, 0.47) | 1346 | 700 | 646 |
| Adolescence peer problems child-rated | 0.47 (0.34, 0.51) | 0.00 (0.00, 0.11) | 0.53 (0.49, 0.57) | 2136 | 1112 | 1024 |
|  |  |  |  |  |  |  |
| Adulthood hyperactivity parent-rated | 0.59 (0.54, 0.64) | 0.00 (0.00, 0.02) | 0.41 (0.36, 0.46) | 1530 | 788 | 742 |
| Adulthood hyperactivity child-rated | 0.34 (0.26, 0.41) | 0.00 (0.00, 0.15) | 0.66 (0.59, 0.74) | 920 | 497 | 423 |
| Adulthood conduct problems parent-rated | 0.52 (0.36, 0.57) | 0.01 (0.00, 0.14) | 0.47 (0.43, 0.53) | 1532 | 789 | 743 |
| Adulthood conduct problems child-rated | 0.26 (0.00, 0.35) | 0.02 (0.00, 0.24) | 0.72 (0.65, 0.80) | 921 | 497 | 424 |
| Adulthood emotional problems parent-rated | 0.53 (0.47, 0.58) | 0.00 (0.00, 0.04) | 0.47 (0.42, 0.52) | 1525 | 785 | 740 |
| Adulthood emotional problems child-rated | 0.06 (0.00, 0.29) | 0.23 (0.04, 0.33) | 0.71 (0.63, 0.78) | 921 | 497 | 424 |
| Adulthood peer problems parent-rated | 0.68 (0.65, 0.72) | 0.00 (0.00, 0.03) | 0.32 (0.28, 0.35) | 1532 | 789 | 743 |
| Adulthood peer problems child-rated | 0.32 (0.08, 0.42) | 0.03 (0.00, 0.22) | 0.65 (0.58, 0.73) | 921 | 497 | 424 |
|  |  |  |  |  |  |  |
| **Poly-E composites** | **h2 (95% CIs)** | **c2 (95% CIs)** | **e2 (95% CIs)** | **N total** | **N MZ** | **N DZ** |
|  |  |  |  |  |  |  |
| Preschool hyperactivity poly-E | 0.31 (0.26, 0.37) | 0.59 (0.54, 0.64) | 0.10 (0.09, 0.11) | 2649 | 1351 | 1298 |
| Preschool conduct problems poly-E | 0.39 (0.33, 0.47) | 0.48 (0.41, 0.54) | 0.13 (0.11, 0.14) | 2651 | 1351 | 1300 |
| Preschool emotional problems poly-E | 0.10 (0.08, 0.12) | 0.86 (0.84, 0.88) | 0.04 (0.04, 0.05) | 2651 | 1351 | 1300 |
| Preschool peer problems poly-E | 0.10 (0.08, 0.13) | 0.85 (0.83, 0.87) | 0.05 (0.04, 0.05) | 2651 | 1351 | 1300 |
|  |  |  |  |  |  |  |
| Childhood hyperactivity poly-E | 0.64 (0.52, 0.77) | 0.22 (0.10, 0.34) | 0.14 (0.12, 0.17) | 2156 | 1123 | 1033 |
| Childhood conduct problems poly-E | 0.58 (0.47, 0.71) | 0.27 (0.15, 0.38) | 0.15 (0.13, 0.17) | 2156 | 1123 | 1033 |
| Childhood emotional problems poly-E | 0.33 (0.20, 0.46) | 0.38 (0.26, 0.49) | 0.29 (0.26, 0.33) | 1837 | 956 | 881 |
| Childhood peer problems poly-E | 0.72 (0.60, 0.75) | 0.00 (0.00, 0.11) | 0.28 (0.25, 0.32) | 1838 | 956 | 882 |
|  |  |  |  |  |  |  |
| Adolescence hyperactivity poly-E | 0.51 (0.44, 0.60) | 0.34 (0.26, 0.42) | 0.14 (0.13, 0.16) | 1530 | 788 | 742 |
| Adolescence conduct problems poly-E | 0.47 (0.39, 0.55) | 0.39 (0.31, 0.46) | 0.15 (0.13, 0.16) | 1532 | 789 | 743 |
| *Note.* h2= genetic influences; c2= shared environmental influences; e2= nonshared environmental influences; CIs= confidence intervals; N= sample size; MZ= monozygotic twin pairs; DZ= dizygotic twin pairs. |  |  |  |  |  |  |

**Table S****5****.** Genetic, shared and nonshared environmental influences on behaviour problem symptoms and poly-E composites (i.e., environmental measures) estimated for females.

| **Behaviour problem symptoms** | **h2 (95% CIs)** | **c2 (95% CIs)** | **e2 (95% CIs)** | **N total** | **N MZ** | **N DZ** |
| --- | --- | --- | --- | --- | --- | --- |
|  |  |  |  |  |  |  |
| Preschool hyperactivity parent-rated | 0.50 (0.46, 0.54) | 0.00 (0.00, 0.01) | 0.50 (0.46, 0.54) | 3127 | 1642 | 1485 |
| Preschool conduct problems parent-rated | 0.57 (0.48, 0.66) | 0.11 (0.03, 0.19) | 0.32 (0.30, 0.34) | 3126 | 1642 | 1484 |
| Preschool emotional problems parent-rated | 0.59 (0.53, 0.62) | 0.00 (0.00, 0.05) | 0.41 (0.38, 0.44) | 3129 | 1642 | 1487 |
| Preschool peer problems parent-rated | 0.59 (0.53, 0.62) | 0.00 (0.00, 0.05) | 0.41 (0.38, 0.44) | 3129 | 1642 | 1485 |
|  |  |  |  |  |  |  |
| Childhood hyperactivity parent-rated | 0.58 (0.54, 0.62) | 0.00 (0.00, 0.01) | 0.42 (0.38, 0.46) | 2863 | 1521 | 1342 |
| Childhood hyperactivity teacher-rated | 0.71 (0.66, 0.73) | 0.00 (0.00, 0.04) | 0.29 (0.27, 0.32) | 2387 | 1269 | 1118 |
| Childhood hyperactivity child-rated | 0.40 (0.29, 0.46) | 0.00 (0.00, 0.09) | 0.60 (0.54, 0.66) | 1240 | 684 | 556 |
| Childhood conduct parent-rated | 0.59 (0.51, 0.67) | 0.16 (0.08, 0.24) | 0.25 (0.23, 0.27) | 2866 | 1523 | 1343 |
| Childhood conduct teacher-rated | 0.58 (0.48, 0.69) | 0.10 (0.00, 0.19) | 0.32 (0.29, 0.35) | 2387 | 1269 | 1118 |
| Childhood conduct child-rated | 0.44 (0.26, 0.53) | 0.04 (0.00, 0.18) | 0.52 (0.47, 0.58) | 1240 | 684 | 556 |
| Childhood emotional problems parent-rated | 0.50 (0.40, 0.60) | 0.15 (0.05, 0.23) | 0.36 (0.33, 0.39) | 2864 | 1523 | 1341 |
| Childhood emotional problems teacher-rated | 0.55 (0.50, 0.58) | 0.00 (0.00, 0.04) | 0.45 (0.42, 0.49) | 2376 | 1262 | 1114 |
| Childhood emotional problems child-rated | 0.49 (0.39, 0.54) | 0.00 (0.00, 0.08) | 0.51 (0.46, 0.56) | 1237 | 683 | 554 |
| Childhood peer problems parent-rated | 0.67 (0.64, 0.69) | 0.00 (0.00, 0.04) | 0.33 (0.31, 0.36) | 2864 | 1523 | 1341 |
| Childhood peer problems teacher-rated | 0.49 (0.38, 0.60) | 0.15 (0.05, 0.25) | 0.36 (0.33, 0.39) | 2383 | 1266 | 1117 |
| Childhood peer problems child-rated | 0.41 (0.22, 0.48) | 0.01 (0.00, 0.17) | 0.58 (0.52, 0.64) | 1239 | 684 | 555 |
|  |  |  |  |  |  |  |
| Adolescence hyperactivity parent-rated | 0.75 (0.72, 0.77) | 0.00 (0.00, 0.01) | 0.25 (0.23, 0.28) | 2562 | 1357 | 1205 |
| Adolescence hyperactivity teacher-rated | 0.59 (0.47, 0.63) | 0.00 (0.00, 0.11) | 0.41 (0.37, 0.45) | 1635 | 865 | 770 |
| Adolescence hyperactivity child-rated | 0.48 (0.43, 0.52) | 0.00 (0.00, 0.02) | 0.52 (0.48, 0.57) | 2545 | 1348 | 1197 |
| Adolescence conduct parent-rated | 0.73 (0.65, 0.81) | 0.06 (0.00, 0.14) | 0.21 (0.19, 0.23) | 2563 | 1357 | 1206 |
| Adolescence conduct teacher-rated | 0.43 (0.28, 0.56) | 0.08 (0.00, 0.20) | 0.49 (0.44, 0.55) | 1636 | 865 | 771 |
| Adolescence conduct child-rated | 0.47 (0.40, 0.51) | 0.00 (0.00, 0.05) | 0.53 (0.49, 0.58) | 2546 | 1348 | 1198 |
| Adolescence emotional problems parent-rated | 0.54 (0.42, 0.63) | 0.06 (0.00, 0.17) | 0.40 (0.36, 0.43) | 2161 | 1159 | 1002 |
| Adolescence emotional problems teacher-rated | 0.44 (0.39, 0.49) | 0.00 (0.00, 0.07) | 0.56 (0.51, 0.61) | 1635 | 865 | 770 |
| Adolescence emotional problems child-rated | 0.44 (0.40, 0.48) | 0.00 (0.00, 0.09) | 0.56 (0.52, 0.60) | 2546 | 1348 | 1198 |
| Adolescence peer problems parent-rated | 0.71 (0.61, 0.76) | 0.03 (0.00, 0.13) | 0.26 (0.23, 0.28) | 2161 | 1341 | 1002 |
| Adolescence peer problems teacher-rated | 0.52 (0.38, 0.60) | 0.03 (0.00, 0.16) | 0.44 (0.40, 0.49) | 1634 | 865 | 769 |
| Adolescence peer problems child-rated | 0.52 (0.41, 0.55) | 0.00 (0.00, 0.09) | 0.48 (0.45, 0.52) | 2546 | 1348 | 1198 |
|  |  |  |  |  |  |  |
| Adulthood hyperactivity parent-rated | 0.58 (0.54, 0.62) | 0.00 (0.00, 0.03) | 0.42 (0.38, 0.46) | 2088 | 1108 | 980 |
| Adulthood hyperactivity child-rated | 0.34 (0.18, 0.40) | 0.01 (0.00, 0.15) | 0.65 (0.60, 0.70) | 1817 | 1004 | 813 |
| Adulthood conduct parent-rated | 0.54 (0.42, 0.60) | 0.02 (0.00, 0.13) | 0.43 (0.40, 0.47) | 2090 | 1109 | 981 |
| Adulthood conduct child-rated | 0.19 (0.01, 0.33) | 0.08 (0.00, 0.22) | 0.73 (0.67, 0.80) | 1817 | 1004 | 813 |
| Adulthood emotional problems parent-rated | 0.54 (0.46, 0.58) | 0.00 (0.00, 0.07) | 0.46 (0.42, 0.50) | 2086 | 1106 | 980 |
| Adulthood emotional problems child-rated | 0.31 (0.15, 0.42) | 0.06 (0.00, 0.19) | 0.63 (0.58, 0.69) | 1817 | 1004 | 813 |
| Adulthood peer problems parent-rated | 0.68 (0.57, 0.71) | 0.00 (0.00, 0.10) | 0.32 (0.29, 0.35) | 2087 | 1107 | 980 |
| Adulthood peer problems child-rated | 0.41 (0.37, 0.46) | 0.00 (0.00, 0.13) | 0.59 (0.54, 0.63) | 1817 | 1004 | 813 |
|  |  |  |  |  |  |  |
| **Poly-E composites** | **h2 (95% CIs)** | **c2 (95% CIs)** | **e2 (95% CIs)** | **N total** | **N MZ** | **N DZ** |
|  |  |  |  |  |  |  |
| Preschool hyperactivity poly-E | 0.26 (0.21, 0.32) | 0.64 (0.58, 0.68) | 0.10 (0.09, 0.11) | 2863 | 1521 | 1342 |
| Preschool conduct problems poly-E | 0.30 (0.24, 0.37) | 0.56 (0.50, 0.62) | 0.14 (0.12, 0.15) | 2866 | 1523 | 1343 |
| Preschool emotional problems poly-E | 0.07 (0.05, 0.09) | 0.88 (0.86, 0.90) | 0.04 (0.04, 0.05) | 2864 | 1523 | 1341 |
| Preschool peer problems poly-E | 0.07 (0.05, 0.09) | 0.88 (0.86, 0.90) | 0.05 (0.04, 0.05) | 2864 | 1523 | 1341 |
|  |  |  |  |  |  |  |
| Childhood hyperactivity poly-E | 0.53 (0.43, 0.65) | 0.32 (0.20, 0.42) | 0.15 (0.13, 0.17) | 2562 | 1357 | 1205 |
| Childhood conduct problems poly-E | 0.58 (0.47, 0.70) | 0.26 (0.14, 0.36) | 0.16 (0.14, 0.19) | 2563 | 1357 | 1206 |
| Childhood emotional problems poly-E | 0.34 (0.21, 0.48) | 0.31 (0.19, 0.43) | 0.35 (0.31, 0.39) | 2161 | 1159 | 1002 |
| Childhood peer problems poly-E | 0.62 (0.48, 0.72) | 0.07 (0.00, 0.20) | 0.31 (0.28, 0.35) | 2161 | 1341 | 1002 |
|  |  |  |  |  |  |  |
| Adolescence hyperactivity poly-E | 0.40 (0.33, 0.46) | 0.46 (0.4, 0.52) | 0.14 (0.13, 0.15) | 2088 | 1108 | 980 |
| Adolescence conduct problems poly-E | 0.36 (0.30, 0.43) | 0.5 (0.44, 0.55) | 0.14 (0.13, 0.15) | 2090 | 1109 | 981 |
| *Note.* h2= genetic influences; c2= shared environmental influences; e2= nonshared environmental influences; CIs= confidence intervals; N= sample size; MZ= monozygotic twin pairs; DZ= dizygotic twin pairs. |  |  |  |  |  |  |

**Table S****6****.** Genetic, shared and nonshared environmental standardised squared bivariate path estimates calculated for the total sample. 

| **Genetic paths** |  |  |  |  |  |  |
| --- | --- | --- | --- | --- | --- | --- |
| **Poly-E & behaviour problem symptoms** | **a11 (95% CIs)** | **a21 (95% CIs)** | **a22 (95% CIs)** | **N total** | **N MZ** | **N DZ** |
|  |  |  |  |  |  |  |
| Preschool poly-E parent-rated & childhood hyperactivity parent-rated | 0.30 (0.27, 0.33) | 0.16 (0.12, 0.21) | 0.33 (0.28, 0.38) | 3947 | 1434 | 2513 |
| Preschool poly-E parent-rated & childhood conduct parent-rated | 0.36 (0.32, 0.40) | 0.21 (0.17, 0.27) | 0.41 (0.35, 0.47) | 3833 | 1387 | 2446 |
| Preschool poly-E parent-rated & childhood emotional problems parent-rated | 0.09 (0.08, 0.11) | 0.01 (0.00, 0.02) | 0.48 (0.42, 0.53) | 3991 | 1451 | 2540 |
| Preschool poly-E parent-rated & childhood peer problems parent-rated | 0.10 (0.08, 0.11) | 0.00 (0.00, 0.02) | 0.60 (0.58, 0.62) | 3991 | 1451 | 2540 |
| Preschool poly-E parent-rated & childhood hyperactivity teacher-rated | 0.30 (0.27, 0.33) | 0.08 (0.05, 0.12) | 0.61 (0.56, 0.64) | 3461 | 1262 | 2199 |
| Preschool poly-E parent-rated & childhood conduct teacher-rated | 0.37 (0.33, 0.41) | 0.08 (0.05, 0.13) | 0.60 (0.55, 0.64) | 3361 | 1221 | 2140 |
| Preschool poly-E parent-rated & childhood emotional problems teacher-rated | 0.10 (0.08, 0.11) | 0.00 (0.00, 0.01) | 0.51 (0.48, 0.53) | 3494 | 1272 | 2222 |
| Preschool poly-E parent-rated & childhood peer problems teacher-rated | 0.10 (0.09, 0.11) | 0.01 (0.00, 0.03) | 0.64 (0.61, 0.66) | 3496 | 1275 | 2221 |
| Preschool poly-E parent-rated & childhood hyperactivity child-rated | 0.30 (0.27, 0.33) | 0.07 (0.03, 0.12) | 0.31 (0.25, 0.37) | 2588 | 969 | 1619 |
| Preschool poly-E parent-rated & childhood conduct child-rated | 0.37 (0.33, 0.41) | 0.07 (0.03, 0.13) | 0.38 (0.26, 0.43) | 2509 | 937 | 1572 |
| Preschool poly-E parent-rated & childhood emotional problems child-rated | 0.10 (0.08, 0.11) | 0.01 (0.00, 0.04) | 0.38 (0.26, 0.42) | 2609 | 975 | 1634 |
| Preschool poly-E parent-rated & childhood peer problems child-rated | 0.10 (0.08, 0.11) | 0.01 (0.00, 0.04) | 0.30 (0.18, 0.41) | 2587 | 971 | 1616 |
|  |  |  |  |  |  |  |
| Childhood poly-E parent-rated & adolescence hyperactivity parent-rated | 0.68 (0.61, 0.75) | 0.25 (0.22, 0.29) | 0.45 (0.41, 0.49) | 2474 | 958 | 1516 |
| Childhood poly-E parent-rated & adolescence conduct parent-rated | 0.64 (0.57, 0.71) | 0.21 (0.16, 0.26) | 0.50 (0.43, 0.56) | 2558 | 976 | 1582 |
| Childhood poly-E parent-rated & adolescence emotional problems parent-rated | 0.33 (0.26, 0.41) | 0.11 (0.04, 0.19) | 0.45 (0.35, 0.52) | 2663 | 1015 | 1648 |
| Childhood poly-E parent-rated & adolescence peer problems parent-rated | 0.60 (0.52, 0.67) | 0.24 (0.19, 0.30) | 0.48 (0.42, 0.54) | 2630 | 1007 | 1623 |
| Childhood poly-E parent-rated & adolescence hyperactivity teacher-rated | 0.70 (0.62, 0.78) | 0.08 (0.05, 0.13) | 0.54 (0.48, 0.58) | 1379 | 547 | 832 |
| Childhood poly-E parent-rated & adolescence conduct teacher-rated | 0.64 (0.57, 0.72) | 0.03 (0.01, 0.06) | 0.59 (0.53, 0.62) | 1423 | 554 | 869 |
| Childhood poly-E parent-rated & adolescence emotional problems teacher-rated | 0.33 (0.25, 0.41) | 0.03 (0.00, 0.11) | 0.39 (0.32, 0.44) | 1559 | 607 | 952 |
| Childhood poly-E parent-rated & adolescence peer problems teacher-rated | 0.62 (0.54, 0.71) | 0.10 (0.06, 0.15) | 0.46 (0.39, 0.50) | 1550 | 605 | 945 |
| Childhood poly-E parent-rated & adolescence hyperactivity child-rated | 0.71 (0.63, 0.79) | 0.09 (0.06, 0.12) | 0.37 (0.33, 0.40) | 2451 | 949 | 1502 |
| Childhood poly-E parent-rated & adolescence conduct child-rated | 0.65 (0.58, 0.73) | 0.05 (0.03, 0.09) | 0.39 (0.36, 0.42) | 2532 | 967 | 1565 |
| Childhood poly-E parent-rated & adolescence emotional problems child-rated | 0.33 (0.25, 0.41) | 0.02 (0.00, 0.07) | 0.37 (0.32, 0.40) | 2840 | 1084 | 1756 |
| Childhood poly-E parent-rated & adolescence peer problems child-rated | 0.63 (0.54, 0.71) | 0.12 (0.08, 0.18) | 0.37 (0.30, 0.41) | 2808 | 1076 | 1732 |
|  |  |  |  |  |  |  |
| Adolescence poly-E parent-rated & adulthood hyperactivity parent-rated | 0.45 (0.41, 0.49) | 0.08 (0.05, 0.11) | 0.49 (0.44, 0.52) | 4036 | 1477 | 2559 |
| Adolescence poly-E parent-rated & adulthood conduct parent-rated | 0.43 (0.39, 0.48) | 0.11 (0.07, 0.16) | 0.44 (0.37, 0.48) | 4039 | 1477 | 2562 |
| Adolescence poly-E parent-rated & adulthood hyperactivity child-rated | 0.45 (0.41, 0.50) | 0.01 (0.00, 0.04) | 0.33 (0.25, 0.37) | 2914 | 1141 | 1773 |
| Adolescence poly-E parent-rated & adulthood conduct child-rated | 0.43 (0.39, 0.48) | 0.04 (0.01, 0.08) | 0.23 (0.12, 0.28) | 2912 | 1140 | 1772 |
| **Shared environmental paths** |  |  |  |  |  |  |
| **Poly-E & behaviour problem symptoms** | **c11 (95% CIs)** | **c21 (95% CIs)** | **c22 (95% CIs)** | **N total** | **N MZ** | **N DZ** |
|  |  |  |  |  |  |  |
| Preschool poly-E parent-rated & childhood hyperactivity parent-rated | 0.60 (0.57, 0.62) | 0.01 (0.01, 0.02) | 0.00 (0.00, 0.00) | 3947 | 1434 | 2513 |
| Preschool poly-E parent-rated & childhood conduct parent-rated | 0.49 (0.46, 0.52) | 0.03 (0.01, 0.05) | 0.11 (0.07, 0.15) | 3833 | 1387 | 2446 |
| Preschool poly-E parent-rated & childhood emotional problems parent-rated | 0.85 (0.82, 0.88) | 0.08 (0.07, 0.10) | 0.07 (0.05, 0.12) | 3991 | 1451 | 2540 |
| Preschool poly-E parent-rated & childhood peer problems parent-rated | 0.85 (0.82, 0.88) | 0.05 (0.03, 0.06) | 0.00 (0.00, 0.01) | 3991 | 1451 | 2540 |
| Preschool poly-E parent-rated & childhood hyperactivity teacher-rated | 0.60 (0.57, 0.63) | 0.00 (0.00, 0.01) | 0.00 (0.00, 0.01) | 3461 | 1262 | 2199 |
| Preschool poly-E parent-rated & childhood conduct teacher-rated | 0.50 (0.46, 0.53) | 0.00 (0.00, 0.01) | 0.00 (0.00, 0.03) | 3361 | 1221 | 2140 |
| Preschool poly-E parent-rated & childhood emotional problems teacher-rated | 0.86 (0.83, 0.89) | 0.01 (0.00, 0.01) | 0.00 (0.00, 0.01) | 3494 | 1272 | 2222 |
| Preschool poly-E parent-rated & childhood peer problems teacher-rated | 0.86 (0.83, 0.89) | 0.01 (0.00, 0.01) | 0.00 (0.00, 0.02) | 3496 | 1275 | 2221 |
| Preschool poly-E parent-rated & childhood hyperactivity child-rated | 0.60 (0.58, 0.63) | 0.00 (0.00, 0.01) | 0.00 (0.00, 0.02) | 2588 | 969 | 1619 |
| Preschool poly-E parent-rated & childhood conduct child-rated | 0.50 (0.47, 0.53) | 0.02 (0.00, 0.04) | 0.01 (0.00, 0.09) | 2509 | 937 | 1572 |
| Preschool poly-E parent-rated & childhood emotional problems child-rated | 0.86 (0.83, 0.89) | 0.04 (0.03, 0.06) | 0.00 (0.00, 0.09) | 2609 | 975 | 1634 |
| Preschool poly-E parent-rated & childhood peer problems child-rated | 0.86 (0.83, 0.89) | 0.03 (0.01, 0.04) | 0.06 (0.00, 0.15) | 2587 | 971 | 1616 |
|  |  |  |  |  |  |  |
| Childhood poly-E & adolescence hyperactivity parent-rated | 0.15 (0.09, 0.20) | 0.00 (0.00, 0.01) | 0.00 (0.00, 0.00) | 2474 | 958 | 1516 |
| Childhood poly-E & adolescence conduct parent-rated | 0.19 (0.13, 0.25) | 0.01 (0.00, 0.03) | 0.07 (0.02, 0.11) | 2558 | 976 | 1582 |
| Childhood poly-E & adolescence emotional problems parent-rated | 0.34 (0.27, 0.40) | 0.02 (0.00, 0.05) | 0.03 (0.00, 0.09) | 2663 | 1015 | 1648 |
| Childhood poly-E & adolescence peer problems parent-rated | 0.09 (0.03, 0.15) | 0.00 (0.00, 0.02) | 0.00 (0.00, 0.02) | 2630 | 1007 | 1623 |
| Childhood poly-E & adolescence hyperactivity teacher-rated | 0.15 (0.08, 0.21) | 0.00 (0.00, 0.01) | 0.00 (0.00, 0.03) | 1379 | 547 | 832 |
| Childhood poly-E & adolescence conduct teacher-rated | 0.20 (0.13, 0.25) | 0.00 (0.00, 0.03) | 0.00 (0.00, 0.04) | 1423 | 554 | 869 |
| Childhood poly-E & adolescence emotional problems teacher-rated | 0.34 (0.28, 0.40) | 0.01 (0.00, 0.03) | 0.00 (0.00, 0.03) | 1559 | 607 | 952 |
| Childhood poly-E & adolescence peer problems teacher-rated | 0.07 (0.00, 0.14) | 0.00 (0.00, 0.02) | 0.00 (0.00, 0.02) | 1550 | 605 | 945 |
| Childhood poly-E & adolescence hyperactivity child-rated | 0.14 (0.08, 0.20) | 0.00 (0.00, 0.01) | 0.00 (0.00, 0.01) | 2451 | 949 | 1502 |
| Childhood poly-E & adolescence conduct child-rated | 0.19 (0.13, 0.25) | 0.01 (0.00, 0.04) | 0.00 (0.00, 0.02) | 2532 | 967 | 1565 |
| Childhood poly-E & adolescence emotional problems child-rated | 0.34 (0.28, 0.40) | 0.01 (0.00, 0.04) | 0.00 (0.00, 0.04) | 2840 | 1084 | 1756 |
| Childhood poly-E & adolescence peer problems child-rated | 0.07 (0.00, 0.14) | 0.00 (0.00, 0.03) | 0.00 (0.00, 0.03) | 2808 | 1076 | 1732 |
|  |  |  |  |  |  |  |
| Adolescence poly-E parent-rated & adulthood hyperactivity parent-rated | 0.41 (0.37, 0.44) | 0.00 (0.00, 0.01) | 0.00 (0.00, 0.01) | 4036 | 1477 | 2559 |
| Adolescence poly-E parent-rated & adulthood conduct parent-rated | 0.43 (0.39, 0.46) | 0.00 (0.00, 0.01) | 0.00 (0.00, 0.05) | 4039 | 1477 | 2562 |
| Adolescence poly-E parent-rated & adulthood hyperactivity child-rated | 0.40 (0.37, 0.44) | 0.00 (0.00, 0.02) | 0.00 (0.00, 0.06) | 2914 | 1141 | 1773 |
| Adolescence poly-E parent-rated & adulthood conduct child-rated | 0.43 (0.39, 0.46) | 0.00 (0.00, 0.01) | 0.00 (0.00, 0.09) | 2912 | 1140 | 1772 |
| **Nonshared environmental paths** |  |  |  |  |  |  |
| **Poly-E & behaviour problem symptoms** | **e11 (95% CIs)** | **e21 (95% CIs)** | **e22 (95% CIs)** | **N total** | **N MZ** | **N DZ** |
|  |  |  |  |  |  |  |
| Preschool poly-E parent-rated & childhood hyperactivity parent-rated | 0.10 (0.09, 0.10) | 0.01 (0.00, 0.02) | 0.47 (0.43, 0.50) | 3947 | 1434 | 2513 |
| Preschool poly-E parent-rated & childhood conduct parent-rated | 0.12 (0.11, 0.14) | 0.01 (0.00, 0.01) | 0.22 (0.21, 0.24) | 3833 | 1387 | 2446 |
| Preschool poly-E parent-rated & childhood emotional problems parent-rated | 0.04 (0.04, 0.05) | 0.00 (0.00, 0.01) | 0.36 (0.34, 0.38) | 3991 | 1451 | 2540 |
| Preschool poly-E parent-rated & childhood peer problems parent-rated | 0.04 (0.04, 0.05) | 0.00 (0.00, 0.01) | 0.33 (0.30, 0.35) | 3991 | 1451 | 2540 |
| Preschool poly-E parent-rated & childhood hyperactivity teacher-rated | 0.10 (0.09, 0.10) | 0.00 (0.00, 0.00) | 0.29 (0.27, 0.31) | 3461 | 1262 | 2199 |
| Preschool poly-E parent-rated & childhood conduct teacher-rated | 0.13 (0.12, 0.14) | 0.00 (0.00, 0.00) | 0.30 (0.28, 0.33) | 3361 | 1221 | 2140 |
| Preschool poly-E parent-rated & childhood emotional problems teacher-rated | 0.04 (0.04, 0.05) | 0.00 (0.00, 0.01) | 0.48 (0.45, 0.52) | 3494 | 1272 | 2222 |
| Preschool poly-E parent-rated & childhood peer problems teacher-rated | 0.04 (0.04, 0.05) | 0.00 (0.00, 0.00) | 0.34 (0.31, 0.36) | 3496 | 1275 | 2221 |
| Preschool poly-E parent-rated & childhood hyperactivity child-rated | 0.10 (0.09, 0.11) | 0.00 (0.00, 0.01) | 0.61 (0.56, 0.66) | 2588 | 969 | 1619 |
| Preschool poly-E parent-rated & childhood conduct child-rated | 0.13 (0.12, 0.14) | 0.00 (0.00, 0.01) | 0.52 (0.47, 0.57) | 2509 | 937 | 1572 |
| Preschool poly-E parent-rated & childhood emotional problems child-rated | 0.04 (0.04, 0.05) | 0.00 (0.00, 0.00) | 0.57 (0.52, 0.62) | 2609 | 975 | 1634 |
| Preschool poly-E parent-rated & childhood peer problems child-rated | 0.05 (0.04, 0.05) | 0.00 (0.00, 0.01) | 0.60 (0.55, 0.66) | 2587 | 971 | 1616 |
|  |  |  |  |  |  |  |
| Childhood poly-E & adolescence hyperactivity parent-rated | 0.14 (0.12, 0.16) | 0.02 (0.01, 0.03) | 0.23 (0.21, 0.25) | 2474 | 958 | 1516 |
| Childhood poly-E & adolescence conduct parent-rated | 0.15 (0.13, 0.16) | 0.01 (0.01, 0.02) | 0.20 (0.18, 0.21) | 2558 | 976 | 1582 |
| Childhood poly-E & adolescence emotional problems parent-rated | 0.33 (0.29, 0.36) | 0.02 (0.01, 0.03) | 0.38 (0.35, 0.41) | 2663 | 1015 | 1648 |
| Childhood poly-E & adolescence peer problems parent-rated | 0.30 (0.27, 0.33) | 0.02 (0.01, 0.03) | 0.24 (0.22, 0.26) | 2630 | 1007 | 1623 |
| Childhood poly-E & adolescence hyperactivity teacher-rated | 0.14 (0.13, 0.16) | 0.01 (0.00, 0.02) | 0.36 (0.33, 0.40) | 1379 | 547 | 832 |
| Childhood poly-E & adolescence conduct teacher-rated | 0.15 (0.13, 0.17) | 0.00 (0.00, 0.01) | 0.38 (0.34, 0.41) | 1423 | 554 | 869 |
| Childhood poly-E & adolescence emotional problems teacher-rated | 0.33 (0.30, 0.36) | 0.00 (0.00, 0.01) | 0.57 (0.53, 0.61) | 1559 | 607 | 952 |
| Childhood poly-E & adolescence peer problems teacher-rated | 0.30 (0.27, 0.33) | 0.00 (0.00, 0.01) | 0.44 (0.40, 0.48) | 1550 | 605 | 945 |
| Childhood poly-E & adolescence hyperactivity child-rated | 0.15 (0.13, 0.16) | 0.01 (0.00, 0.02) | 0.53 (0.49, 0.56) | 2451 | 949 | 1502 |
| Childhood poly-E & adolescence conduct child-rated | 0.15 (0.13, 0.17) | 0.02 (0.01, 0.03) | 0.52 (0.49, 0.56) | 2532 | 967 | 1565 |
| Childhood poly-E & adolescence emotional problems child-rated | 0.33 (0.29, 0.36) | 0.01 (0.00, 0.02) | 0.58 (0.55, 0.62) | 2840 | 1084 | 1756 |
| Childhood poly-E & adolescence peer problems child-rated | 0.29 (0.27, 0.33) | 0.00 (0.00, 0.01) | 0.50 (0.47, 0.53) | 2808 | 1076 | 1732 |
|  |  |  |  |  |  |  |
| Adolescence poly-E parent-rated & adulthood hyperactivity parent-rated | 0.14 (0.13, 0.15) | 0.00 (0.00, 0.01) | 0.41 (0.38, 0.45) | 4036 | 1477 | 2559 |
| Adolescence poly-E parent-rated & adulthood conduct parent-rated | 0.14 (0.13, 0.15) | 0.00 (0.00, 0.01) | 0.45 (0.41, 0.48) | 4039 | 1477 | 2562 |
| Adolescence poly-E parent-rated & adulthood hyperactivity child-rated | 0.14 (0.13, 0.15) | 0.00 (0.00, 0.00) | 0.65 (0.61, 0.70) | 2914 | 1141 | 1773 |
| Adolescence poly-E parent-rated & adulthood conduct child-rated | 0.14 (0.13, 0.15) | 0.00 (0.00, 0.01) | 0.73 (0.68, 0.78) | 2912 | 1140 | 1772 |
| *Note.* a= genetic path; c= shared environmental path; e= nonshared environmental path; CIs= confidence intervals; N= sample size; MZ= monozygotic twin pairs; DZ= dizygotic twin pairs. |  |  |  |  |  |  |

**Table S****7****.** Genetic, shared and nonshared environmental squared bivariate path estimates calculated for males.

| **Genetic paths** |  |  |  |  |  |  |
| --- | --- | --- | --- | --- | --- | --- |
| **Poly-E & behaviour problem symptoms** | **a11 (95% CIs)** | **a21 (95% CIs)** | **a22 (95% CIs)** | **N total** | **N MZ** | **N DZ** |
|  |  |  |  |  |  |  |
| Preschool poly-E parent-rated & childhood hyperactivity parent-rated | 0.31 (0.26, 0.38) | 0.16 (0.10, 0.24) | 0.34 (0.27, 0.40) | 1301 | 658 | 643 |
| Preschool poly-E parent-rated & childhood conduct parent-rated | 0.39 (0.32, 0.48) | 0.24 (0.15, 0.35) | 0.33 (0.24, 0.43) | 1269 | 638 | 631 |
| Preschool poly-E parent-rated & childhood emotional problems parent-rated | 0.10 (0.08, 0.12) | 0.00 (0.00, 0.02) | 0.53 (0.43, 0.58) | 1321 | 666 | 655 |
| Preschool poly-E parent-rated & childhood peer problems parent-rated | 0.11 (0.08, 0.13) | 0.01 (0.00, 0.05) | 0.51 (0.47, 0.54) | 1321 | 666 | 655 |
| Preschool poly-E parent-rated & childhood hyperactivity teacher-rated | 0.31 (0.25, 0.37) | 0.08 (0.03, 0.16) | 0.50 (0.44, 0.54) | 1138 | 573 | 565 |
| Preschool poly-E parent-rated & childhood conduct teacher-rated | 0.39 (0.32, 0.48) | 0.11 (0.05, 0.20) | 0.41 (0.33, 0.46) | 1107 | 555 | 552 |
| Preschool poly-E parent-rated & childhood emotional problems teacher-rated | 0.10 (0.08, 0.13) | 0.00 (0.00, 0.05) | 0.49 (0.44, 0.53) | 1151 | 577 | 574 |
| Preschool poly-E parent-rated & childhood peer problems teacher-rated | 0.11 (0.08, 0.13) | 0.01 (0.00, 0.05) | 0.57 (0.50, 0.60) | 1151 | 579 | 572 |
| Preschool poly-E parent-rated & childhood hyperactivity child-rated | 0.31 (0.25, 0.37) | 0.07 (0.01, 0.17) | 0.32 (0.20, 0.39) | 831 | 433 | 398 |
| Preschool poly-E parent-rated & childhood conduct child-rated | 0.40 (0.32, 0.48) | 0.05 (0.00, 0.14) | 0.27 (0.10, 0.40) | 809 | 419 | 390 |
| Preschool poly-E parent-rated & childhood emotional problems child-rated | 0.10 (0.08, 0.13) | 0.06 (0.00, 0.18) | 0.03 (0.00, 0.24) | 841 | 435 | 406 |
| Preschool poly-E parent-rated & childhood peer problems child-rated | 0.10 (0.08, 0.13) | 0.00 (0.00, 0.07) | 0.22 (0.02, 0.39) | 833 | 432 | 401 |
|  |  |  |  |  |  |  |
| Childhood poly-E parent-rated & adolescence hyperactivity parent-rated | 0.56 (0.45, 0.67) | 0.27 (0.21, 0.34) | 0.37 (0.33, 0.44) | 762 | 417 | 345 |
| Childhood poly-E parent-rated & adolescence conduct parent-rated | 0.54 (0.43, 0.68) | 0.25 (0.15, 0.36) | 0.47 (0.36, 0.54) | 781 | 420 | 361 |
| Childhood poly-E parent-rated & adolescence emotional problems parent-rated | 0.32 (0.20, 0.46) | 0.03 (0.00, 0.12) | 0.53 (0.46, 0.58) | 824 | 435 | 389 |
| Childhood poly-E parent-rated & adolescence peer problems parent-rated | 0.63 (0.52, 0.69) | 0.22 (0.17, 0.30) | 0.42 (0.33, 0.47) | 813 | 432 | 381 |
| Childhood poly-E parent-rated & adolescence hyperactivity teacher-rated | 0.57 (0.46, 0.71) | 0.12 (0.05, 0.22) | 0.38 (0.27, 0.45) | 397 | 220 | 177 |
| Childhood poly-E parent-rated & adolescence conduct teacher-rated | 0.57 (0.45, 0.71) | 0.08 (0.01, 0.19) | 0.42 (0.30, 0.47) | 407 | 222 | 185 |
| Childhood poly-E parent-rated & adolescence emotional problems teacher-rated | 0.31 (0.19, 0.44) | 0.07 (0.00, 0.27) | 0.35 (0.13, 0.43) | 450 | 246 | 204 |
| Childhood poly-E parent-rated & adolescence peer problems teacher-rated | 0.63 (0.52, 0.70) | 0.14 (0.07, 0.26) | 0.33 (0.17, 0.44) | 452 | 246 | 206 |
| Childhood poly-E parent-rated & adolescence hyperactivity child-rated | 0.55 (0.44, 0.67) | 0.13 (0.07, 0.22) | 0.31 (0.19, 0.39) | 752 | 410 | 342 |
| Childhood poly-E parent-rated & adolescence conduct child-rated | 0.58 (0.45, 0.72) | 0.05 (0.01, 0.13) | 0.35 (0.28, 0.39) | 771 | 413 | 358 |
| Childhood poly-E parent-rated & adolescence emotional problems child-rated | 0.32 (0.20, 0.45) | 0.02 (0.00, 0.12) | 0.39 (0.24, 0.44) | 874 | 465 | 409 |
| Childhood poly-E parent-rated & adolescence peer problems child-rated | 0.66 (0.55, 0.70) | 0.10 (0.06, 0.14) | 0.35 (0.24, 0.40) | 865 | 462 | 403 |
|  |  |  |  |  |  |  |
| Adolescence poly-E parent-rated & adulthood hyperactivity parent-rated | 0.49 (0.41, 0.58) | 0.09 (0.04, 0.15) | 0.48 (0.40, 0.54) | 1156 | 598 | 558 |
| Adolescence poly-E parent-rated & adulthood conduct parent-rated | 0.46 (0.38, 0.55) | 0.14 (0.06, 0.25) | 0.44 (0.25, 0.53) | 1156 | 597 | 559 |
| Adolescence poly-E parent-rated & adulthood hyperactivity child-rated | 0.50 (0.41, 0.59) | 0.05 (0.00, 0.14) | 0.29 (0.20, 0.38) | 706 | 380 | 326 |
| Adolescence poly-E parent-rated & adulthood conduct child-rated | 0.46 (0.38, 0.55) | 0.02 (0.00, 0.12) | 0.24 (0.00, 0.32) | 706 | 379 | 327 |
| **Shared environmental paths** |  |  |  |  |  |  |
| **Poly-E & behaviour problem symptoms** | **c11 (95% CIs)** | **c21 (95% CIs)** | **c22 (95% CIs)** | **N total** | **N MZ** | **N DZ** |
|  |  |  |  |  |  |  |
| Preschool poly-E parent-rated & childhood hyperactivity parent-rated | 0.58 (0.53, 0.63) | 0.03 (0.01, 0.05) | 0.00 (0.00, 0.01) | 1301 | 658 | 643 |
| Preschool poly-E parent-rated & childhood conduct parent-rated | 0.47 (0.40, 0.53) | 0.03 (0.00, 0.07) | 0.10 (0.03, 0.16) | 1269 | 638 | 631 |
| Preschool poly-E parent-rated & childhood emotional problems parent-rated | 0.86 (0.81, 0.91) | 0.12 (0.08, 0.16) | 0.00 (0.00, 0.10) | 1321 | 666 | 655 |
| Preschool poly-E parent-rated & childhood peer problems parent-rated | 0.86 (0.81, 0.91) | 0.04 (0.02, 0.07) | 0.00 (0.00, 0.01) | 1321 | 666 | 655 |
| Preschool poly-E parent-rated & childhood hyperactivity teacher-rated | 0.59 (0.54, 0.64) | 0.00 (0.00, 0.02) | 0.00 (0.00, 0.03) | 1138 | 573 | 565 |
| Preschool poly-E parent-rated & childhood conduct teacher-rated | 0.48 (0.41, 0.54) | 0.00 (0.00, 0.01) | 0.00 (0.00, 0.07) | 1107 | 555 | 552 |
| Preschool poly-E parent-rated & childhood emotional problems teacher-rated | 0.87 (0.82, 0.92) | 0.01 (0.00, 0.02) | 0.00 (0.00, 0.03) | 1151 | 577 | 574 |
| Preschool poly-E parent-rated & childhood peer problems teacher-rated | 0.86 (0.81, 0.91) | 0.01 (0.00, 0.02) | 0.00 (0.00, 0.06) | 1151 | 579 | 572 |
| Preschool poly-E parent-rated & childhood hyperactivity child-rated | 0.59 (0.54, 0.64) | 0.00 (0.00, 0.03) | 0.00 (0.00, 0.05) | 831 | 433 | 398 |
| Preschool poly-E parent-rated & childhood conduct child-rated | 0.48 (0.41, 0.54) | 0.04 (0.00, 0.11) | 0.08 (0.00, 0.21) | 809 | 419 | 390 |
| Preschool poly-E parent-rated & childhood emotional problems child-rated | 0.87 (0.82, 0.92) | 0.03 (0.01, 0.06) | 0.24 (0.07, 0.32) | 841 | 435 | 406 |
| Preschool poly-E parent-rated & childhood peer problems child-rated | 0.86 (0.81, 0.91) | 0.04 (0.02, 0.08) | 0.10 (0.00, 0.26) | 833 | 432 | 401 |
|  |  |  |  |  |  |  |
| Childhood poly-E & adolescence hyperactivity parent-rated | 0.20 (0.10, 0.28) | 0.00 (0.00, 0.01) | 0.00 (0.00, 0.01) | 762 | 417 | 345 |
| Childhood poly-E & adolescence conduct parent-rated | 0.28 (0.17, 0.38) | 0.01 (0.00, 0.06) | 0.00 (0.00, 0.07) | 781 | 420 | 361 |
| Childhood poly-E & adolescence emotional problems parent-rated | 0.37 (0.25, 0.48) | 0.06 (0.01, 0.14) | 0.00 (0.00, 0.06) | 824 | 435 | 389 |
| Childhood poly-E & adolescence peer problems parent-rated | 0.01 (0.00, 0.11) | 0.00 (0.00, 0.03) | 0.00 (0.00, 0.03) | 813 | 432 | 381 |
| Childhood poly-E & adolescence hyperactivity teacher-rated | 0.20 (0.09, 0.30) | 0.00 (0.00, 0.04) | 0.00 (0.00, 0.04) | 397 | 220 | 177 |
| Childhood poly-E & adolescence conduct teacher-rated | 0.27 (0.24, 0.38) | 0.00 (0.00, 0.06) | 0.00 (0.00, 0.07) | 407 | 222 | 185 |
| Childhood poly-E & adolescence emotional problems teacher-rated | 0.38 (0.26, 0.48) | 0.00 (0.00, 0.06) | 0.00 (0.00, 0.08) | 450 | 246 | 204 |
| Childhood poly-E & adolescence peer problems teacher-rated | 0.02 (0.00, 0.12) | 0.03 (0.00, 0.13) | 0.00 (0.00, 0.11) | 452 | 246 | 206 |
| Childhood poly-E & adolescence hyperactivity child-rated | 0.22 (0.12, 0.32) | 0.00 (0.00, 0.04) | 0.00 (0.00, 0.04) | 752 | 410 | 342 |
| Childhood poly-E & adolescence conduct child-rated | 0.27 (0.14, 0.37) | 0.02 (0.00, 0.08) | 0.00 (0.00, 0.05) | 771 | 413 | 358 |
| Childhood poly-E & adolescence emotional problems child-rated | 0.37 (0.26, 0.48) | 0.02 (0.00, 0.08) | 0.00 (0.00, 0.11) | 874 | 465 | 409 |
| Childhood poly-E & adolescence peer problems child-rated | 0.00 (0.00, 0.09) | 0.00 (0.00, 0.10) | 0.00 (0.00, 0.09) | 865 | 462 | 403 |
|  |  |  |  |  |  |  |
| Adolescence poly-E parent-rated & adulthood hyperactivity parent-rated | 0.35 (0.27, 0.41) | 0.00 (0.00, 0.01) | 0.00 (0.00, 0.02) | 1156 | 598 | 558 |
| Adolescence poly-E parent-rated & adulthood conduct parent-rated | 0.39 (0.31, 0.45) | 0.00 (0.00, 0.02) | 0.00 (0.00, 0.14) | 1156 | 597 | 559 |
| Adolescence poly-E parent-rated & adulthood hyperactivity child-rated | 0.34 (0.26, 0.41) | 0.00 (0.00, 0.06) | 0.00 (0.00, 0.13) | 706 | 380 | 326 |
| Adolescence poly-E parent-rated & adulthood conduct child-rated | 0.38 (0.31, 0.45) | 0.00 (0.00, 0.06) | 0.01 (0.00, 0.21) | 706 | 379 | 327 |
| **Nonshared environmental paths** |  |  |  |  |  |  |
| **Poly-E & behaviour problem symptoms** | **e11 (95% CIs)** | **e21 (95% CIs)** | **e22 (95% CIs)** | **N total** | **N MZ** | **N DZ** |
|  |  |  |  |  |  |  |
| Preschool poly-E parent-rated & childhood hyperactivity parent-rated | 0.10 (0.08, 0.11) | 0.01 (0.00, 0.03) | 0.36 (0.32, 0.41) | 1301 | 658 | 643 |
| Preschool poly-E parent-rated & childhood conduct parent-rated | 0.12 (0.10, 0.14) | 0.00 (0.00, 0.01) | 0.18 (0.16, 0.21) | 1269 | 638 | 631 |
| Preschool poly-E parent-rated & childhood emotional problems parent-rated | 0.04 (0.04, 0.05) | 0.00 (0.00, 0.01) | 0.37 (0.33, 0.41) | 1321 | 666 | 655 |
| Preschool poly-E parent-rated & childhood peer problems parent-rated | 0.05 (0.04, 0.05) | 0.00 (0.00, 0.01) | 0.31 (0.28, 0.35) | 1321 | 666 | 655 |
| Preschool poly-E parent-rated & childhood hyperactivity teacher-rated | 0.10 (0.08, 0.11) | 0.00 (0.00, 0.00) | 0.22 (0.19, 0.24) | 1138 | 573 | 565 |
| Preschool poly-E parent-rated & childhood conduct teacher-rated | 0.13 (0.11, 0.15) | 0.00 (0.00, 0.00) | 0.20 (0.17, 0.22) | 1107 | 555 | 552 |
| Preschool poly-E parent-rated & childhood emotional problems teacher-rated | 0.04 (0.04, 0.05) | 0.00 (0.00, 0.01) | 0.49 (0.44, 0.55) | 1151 | 577 | 574 |
| Preschool poly-E parent-rated & childhood peer problems teacher-rated | 0.05 (0.04, 0.05) | 0.00 (0.00, 0.00) | 0.28 (0.25, 0.32) | 1151 | 579 | 572 |
| Preschool poly-E parent-rated & childhood hyperactivity child-rated | 0.10 (0.08, 0.11) | 0.00 (0.00, 0.01) | 0.54 (0.47, 0.63) | 831 | 433 | 398 |
| Preschool poly-E parent-rated & childhood conduct child-rated | 0.13 (0.11, 0.15) | 0.00 (0.00, 0.01) | 0.46 (0.39, 0.53) | 809 | 419 | 390 |
| Preschool poly-E parent-rated & childhood emotional problems child-rated | 0.04 (0.04, 0.05) | 0.00 (0.00, 0.01) | 0.68 (0.58, 0.77) | 841 | 435 | 406 |
| Preschool poly-E parent-rated & childhood peer problems child-rated | 0.05 (0.04, 0.05) | 0.00 (0.00, 0.01) | 0.61 (0.52, 0.70) | 833 | 432 | 401 |
|  |  |  |  |  |  |  |
| Childhood poly-E & adolescence hyperactivity parent-rated | 0.12 (0.10, 0.15) | 0.03 (0.01, 0.04) | 0.19 (0.16, 0.22) | 762 | 417 | 345 |
| Childhood poly-E & adolescence conduct parent-rated | 0.14 (0.11, 0.17) | 0.01 (0.00, 0.02) | 0.20 (0.18, 0.23) | 781 | 420 | 361 |
| Childhood poly-E & adolescence emotional problems parent-rated | 0.29 (0.24, 0.34) | 0.02 (0.01, 0.05) | 0.42 (0.36, 0.47) | 824 | 435 | 389 |
| Childhood poly-E & adolescence peer problems parent-rated | 0.26 (0.22, 0.31) | 0.03 (0.02, 0.05) | 0.20 (0.17, 0.23) | 813 | 432 | 381 |
| Childhood poly-E & adolescence hyperactivity teacher-rated | 0.13 (0.11, 0.16) | 0.01 (0.00, 0.03) | 0.26 (0.22, 0.30) | 397 | 220 | 177 |
| Childhood poly-E & adolescence conduct teacher-rated | 0.15 (0.12, 0.18) | 0.00 (0.00, 0.02) | 0.25 (0.21, 0.28) | 407 | 222 | 185 |
| Childhood poly-E & adolescence emotional problems teacher-rated | 0.29 (0.24, 0.34) | 0.00 (0.00, 0.02) | 0.53 (0.47, 0.60) | 450 | 246 | 204 |
| Childhood poly-E & adolescence peer problems teacher-rated | 0.26 (0.22, 0.31) | 0.00 (0.00, 0.02) | 0.36 (0.31, 0.41) | 452 | 246 | 206 |
| Childhood poly-E & adolescence hyperactivity child-rated | 0.13 (0.11, 0.16) | 0.03 (0.01, 0.05) | 0.45 (0.40, 0.50) | 752 | 410 | 342 |
| Childhood poly-E & adolescence conduct child-rated | 0.15 (0.12, 0.18) | 0.02 (0.01, 0.04) | 0.44 (0.39, 0.49) | 771 | 413 | 358 |
| Childhood poly-E & adolescence emotional problems child-rated | 0.29 (0.24, 0.34) | 0.00 (0.00, 0.01) | 0.72 (0.65, 0.79) | 874 | 465 | 409 |
| Childhood poly-E & adolescence peer problems child-rated | 0.26 (0.22, 0.31) | 0.01 (0.00, 0.02) | 0.50 (0.45, 0.55) | 865 | 462 | 403 |
|  |  |  |  |  |  |  |
| Adolescence poly-E parent-rated & adulthood hyperactivity parent-rated | 0.14 (0.12, 0.16) | 0.00 (0.00, 0.01) | 0.39 (0.33, 0.45) | 1156 | 598 | 558 |
| Adolescence poly-E parent-rated & adulthood conduct parent-rated | 0.14 (0.12, 0.16) | 0.00 (0.00, 0.01) | 0.53 (0.47, 0.60) | 1156 | 597 | 559 |
| Adolescence poly-E parent-rated & adulthood hyperactivity child-rated | 0.14 (0.12, 0.16) | 0.00 (0.00, 0.01) | 0.66 (0.57, 0.75) | 706 | 380 | 326 |
| Adolescence poly-E parent-rated & adulthood conduct child-rated | 0.14 (0.12, 0.17) | 0.01 (0.00, 0.02) | 0.68 (0.59, 0.77) | 706 | 379 | 327 |
| *Note.* a= genetic path; c= shared environmental path; e= nonshared environmental path; CIs= confidence intervals; N= sample size; MZ= monozygotic twin pairs; DZ= dizygotic twin pairs. |  |  |  |  |  |  |

**Table S****8****.** Genetic, shared and nonshared environmental squared bivariate path estimates calculated for females.

| **Genetic paths** |  |  |  |  |  |  |
| --- | --- | --- | --- | --- | --- | --- |
| **Poly-E & behaviour problem symptoms** | **a11 (95% CIs)** | **a21 (95% CIs)** | **a22 (95% CIs)** | **N total** | **N MZ** | **N DZ** |
|  |  |  |  |  |  |  |
| Preschool poly-E parent-rated & childhood hyperactivity parent-rated | 0.28 (0.22, 0.34) | 0.16 (0.09, 0.24) | 0.45 (0.37, 0.52) | 1432 | 776 | 656 |
| Preschool poly-E parent-rated & childhood conduct parent-rated | 0.30 (0.24, 0.38) | 0.24 (0.15, 0.35) | 0.42 (0.29, 0.54) | 1388 | 749 | 639 |
| Preschool poly-E parent-rated & childhood emotional problems parent-rated | 0.07 (0.05, 0.10) | 0.00 (0.00, 0.03) | 0.49 (0.39, 0.58) | 1446 | 785 | 661 |
| Preschool poly-E parent-rated & childhood peer problems parent-rated | 0.07 (0.05, 0.09) | 0.01 (0.00, 0.05) | 0.76 (0.71, 0.81) | 1446 | 785 | 661 |
| Preschool poly-E parent-rated & childhood hyperactivity teacher-rated | 0.28 (0.22, 0.34) | 0.10 (0.04, 0.19) | 0.79 (0.67, 0.88) | 1265 | 689 | 576 |
| Preschool poly-E parent-rated & childhood conduct teacher-rated | 0.31 (0.24, 0.39) | 0.08 (0.02, 0.18) | 0.75 (0.57, 0.93) | 1227 | 666 | 561 |
| Preschool poly-E parent-rated & childhood emotional problems teacher-rated | 0.07 (0.06, 0.10) | 0.00 (0.00, 0.05) | 0.50 (0.44, 0.53) | 1273 | 695 | 578 |
| Preschool poly-E parent-rated & childhood peer problems teacher-rated | 0.07 (0.05, 0.10) | 0.00 (0.00, 0.04) | 0.57 (0.44, 0.71) | 1276 | 696 | 580 |
| Preschool poly-E parent-rated & childhood hyperactivity child-rated | 0.28 (0.22, 0.35) | 0.10 (0.02, 0.22) | 0.33 (0.17, 0.44) | 958 | 536 | 422 |
| Preschool poly-E parent-rated & childhood conduct child-rated | 0.31 (0.24, 0.39) | 0.07 (0.01, 0.19) | 0.40 (0.19, 0.51) | 927 | 518 | 409 |
| Preschool poly-E parent-rated & childhood emotional problems child-rated | 0.07 (0.06, 0.10) | 0.02 (0.00, 0.10) | 0.37 (0.26, 0.44) | 964 | 540 | 424 |
| Preschool poly-E parent-rated & childhood peer problems child-rated | 0.07 (0.05, 0.10) | 0.02 (0.00, 0.11) | 0.38 (0.19, 0.44) | 962 | 539 | 423 |
|  |  |  |  |  |  |  |
| Childhood poly-E parent-rated & adolescence hyperactivity parent-rated | 0.59 (0.48, 0.72) | 0.28 (0.20, 0.37) | 0.53 (0.44, 0.61) | 972 | 541 | 431 |
| Childhood poly-E parent-rated & adolescence conduct parent-rated | 0.59 (0.47, 0.73) | 0.14 (0.07, 0.24) | 0.65 (0.54, 0.73) | 1004 | 556 | 448 |
| Childhood poly-E parent-rated & adolescence emotional problems parent-rated | 0.35 (0.22, 0.49) | 0.26 (0.10, 0.49) | 0.27 (0.08, 0.44) | 1043 | 580 | 463 |
| Childhood poly-E parent-rated & adolescence peer problems parent-rated | 0.65 (0.50, 0.75) | 0.27 (0.15, 0.38) | 0.52 (0.36, 0.68) | 1029 | 575 | 454 |
| Childhood poly-E parent-rated & adolescence hyperactivity teacher-rated | 0.59 (0.47, 0.73) | 0.05 (0.00, 0.15) | 0.79 (0.58, 0.86) | 570 | 327 | 243 |
| Childhood poly-E parent-rated & adolescence conduct teacher-rated | 0.61 (0.48, 0.75) | 0.00 (0.00, 0.03) | 0.69 (0.44, 0.87) | 587 | 332 | 255 |
| Childhood poly-E parent-rated & adolescence emotional problems teacher-rated | 0.34 (0.21, 0.49) | 0.03 (0.00, 0.15) | 0.42 (0.27, 0.48) | 637 | 361 | 276 |
| Childhood poly-E parent-rated & adolescence peer problems teacher-rated | 0.66 (0.50, 0.77) | 0.07 (0.01, 0.19) | 0.56 (0.35, 0.67) | 627 | 359 | 268 |
| Childhood poly-E parent-rated & adolescence hyperactivity child-rated | 0.61 (0.48, 0.75) | 0.10 (0.04, 0.18) | 0.40 (0.32, 0.46) | 967 | 539 | 428 |
| Childhood poly-E parent-rated & adolescence conduct child-rated | 0.61 (0.48, 0.75) | 0.05 (0.01, 0.12) | 0.43 (0.36, 0.48) | 998 | 554 | 444 |
| Childhood poly-E parent-rated & adolescence emotional problems child-rated | 0.34 (0.21, 0.49) | 0.02 (0.00, 0.12) | 0.36 (0.26, 0.40) | 1115 | 619 | 496 |
| Childhood poly-E parent-rated & adolescence peer problems child-rated | 0.64 (0.50, 0.75) | 0.21 (0.11, 0.34) | 0.32 (0.14, 0.44) | 1100 | 614 | 486 |
|  |  |  |  |  |  |  |
| Adolescence poly-E parent-rated & adulthood hyperactivity parent-rated | 0.41 (0.34, 0.49) | 0.06 (0.02, 0.11) | 0.56 (0.50, 0.61) | 1625 | 879 | 746 |
| Adolescence poly-E parent-rated & adulthood conduct parent-rated | 0.38 (0.31, 0.45) | 0.07 (0.02, 0.15) | 0.44 (0.32, 0.49) | 1625 | 880 | 745 |
| Adolescence poly-E parent-rated & adulthood hyperactivity child-rated | 0.41 (0.34, 0.49) | 0.02 (0.00, 0.09) | 0.32 (0.15, 0.39) | 1359 | 761 | 598 |
| Adolescence poly-E parent-rated & adulthood conduct child-rated | 0.38 (0.31, 0.46) | 0.05 (0.01, 0.14) | 0.14 (0.00, 0.30) | 1358 | 761 | 597 |
| **Shared environmental paths** |  |  |  |  |  |  |
| **Poly-E & behaviour problem symptoms** | **c11 (95% CIs)** | **c21 (95% CIs)** | **c22 (95% CIs)** | **N total** | **N MZ** | **N DZ** |
|  |  |  |  |  |  |  |
| Preschool poly-E parent-rated & childhood hyperactivity parent-rated | 0.67 (0.62, 0.73) | 0.01 (0.00, 0.03) | 0.00 (0.00, 0.01) | 1432 | 776 | 656 |
| Preschool poly-E parent-rated & childhood conduct parent-rated | 0.58 (0.52, 0.64) | 0.02 (0.00, 0.05) | 0.18 (0.09, 0.26) | 1388 | 749 | 639 |
| Preschool poly-E parent-rated & childhood emotional problems parent-rated | 0.92 (0.86, 0.98) | 0.08 (0.05, 0.11) | 0.06 (0.00, 0.15) | 1446 | 785 | 661 |
| Preschool poly-E parent-rated & childhood peer problems parent-rated | 0.92 (0.86, 0.97) | 0.05 (0.03, 0.08) | 0.00 (0.00, 0.03) | 1446 | 785 | 661 |
| Preschool poly-E parent-rated & childhood hyperactivity teacher-rated | 0.67 (0.62, 0.73) | 0.00 (0.00, 0.01) | 0.00 (0.00, 0.05) | 1265 | 689 | 576 |
| Preschool poly-E parent-rated & childhood conduct teacher-rated | 0.58 (0.52, 0.64) | 0.00 (0.00, 0.03) | 0.14 (0.00, 0.28) | 1227 | 666 | 561 |
| Preschool poly-E parent-rated & childhood emotional problems teacher-rated | 0.93 (0.87, 0.98) | 0.01 (0.00, 0.02) | 0.00 (0.00, 0.04) | 1273 | 695 | 578 |
| Preschool poly-E parent-rated & childhood peer problems teacher-rated | 0.92 (0.87, 0.98) | 0.01 (0.00, 0.02) | 0.17 (0.06, 0.29) | 1276 | 696 | 580 |
| Preschool poly-E parent-rated & childhood hyperactivity child-rated | 0.67 (0.62, 0.73) | 0.00 (0.00, 0.02) | 0.00 (0.00, 0.11) | 958 | 536 | 422 |
| Preschool poly-E parent-rated & childhood conduct child-rated | 0.58 (0.52, 0.64) | 0.02 (0.00, 0.07) | 0.04 (0.00, 0.19) | 927 | 518 | 409 |
| Preschool poly-E parent-rated & childhood emotional problems child-rated | 0.93 (0.87, 0.98) | 0.07 (0.04, 0.11) | 0.00 (0.00, 0.05) | 964 | 540 | 424 |
| Preschool poly-E parent-rated & childhood peer problems child-rated | 0.92 (0.87, 0.98) | 0.02 (0.01, 0.05) | 0.00 (0.00, 0.15) | 962 | 539 | 423 |
|  |  |  |  |  |  |  |
| Childhood poly-E & adolescence hyperactivity parent-rated | 0.32 (0.21, 0.41) | 0.00 (0.00, 0.02) | 0.00 (0.00, 0.01) | 972 | 541 | 431 |
| Childhood poly-E & adolescence conduct parent-rated | 0.27 (0.16, 0.38) | 0.04 (0.00, 0.12) | 0.03 (0.00, 0.11) | 1004 | 556 | 448 |
| Childhood poly-E & adolescence emotional problems parent-rated | 0.29 (0.17, 0.41) | 0.01 (0.00, 0.08) | 0.05 (0.00, 0.16) | 1043 | 580 | 463 |
| Childhood poly-E & adolescence peer problems parent-rated | 0.07 (0.00, 0.20) | 0.04 (0.00, 0.13) | 0.00 (0.00, 0.14) | 1029 | 575 | 454 |
| Childhood poly-E & adolescence hyperactivity teacher-rated | 0.34 (0.22, 0.45) | 0.00 (0.00, 0.07) | 0.00 (0.00, 0.16) | 570 | 327 | 243 |
| Childhood poly-E & adolescence conduct teacher-rated | 0.27 (0.15, 0.38) | 0.04 (0.00, 0.16) | 0.07 (0.00, 0.28) | 587 | 332 | 255 |
| Childhood poly-E & adolescence emotional problems teacher-rated | 0.30 (0.18, 0.42) | 0.00 (0.00, 0.04) | 0.00 (0.00, 0.07) | 637 | 361 | 276 |
| Childhood poly-E & adolescence peer problems teacher-rated | 0.07 (0.00, 0.20) | 0.00 (0.00, 0.15) | 0.03 (0.00, 0.19) | 627 | 359 | 268 |
| Childhood poly-E & adolescence hyperactivity child-rated | 0.33 (0.20, 0.43) | 0.01 (0.00, 0.04) | 0.00 (0.00, 0.02) | 967 | 539 | 428 |
| Childhood poly-E & adolescence conduct child-rated | 0.27 (0.15, 0.37) | 0.02 (0.00, 0.08) | 0.00 (0.00, 0.05) | 998 | 554 | 444 |
| Childhood poly-E & adolescence emotional problems child-rated | 0.30 (0.18, 0.42) | 0.01 (0.00, 0.06) | 0.00 (0.00, 0.07) | 1115 | 619 | 496 |
| Childhood poly-E & adolescence peer problems child-rated | 0.09 (0.00, 0.21) | 0.02 (0.00, 0.10) | 0.00 (0.00, 0.10) | 1100 | 614 | 486 |
|  |  |  |  |  |  |  |
| Adolescence poly-E parent-rated & adulthood hyperactivity parent-rated | 0.49 (0.42, 0.55) | 0.00 (0.00, 0.02) | 0.00 (0.00, 0.03) | 1625 | 879 | 746 |
| Adolescence poly-E parent-rated & adulthood conduct parent-rated | 0.52 (0.46, 0.57) | 0.02 (0.00, 0.06) | 0.01 (0.00, 0.10) | 1625 | 880 | 745 |
| Adolescence poly-E parent-rated & adulthood hyperactivity child-rated | 0.49 (0.42, 0.55) | 0.00 (0.00, 0.02) | 0.01 (0.00, 0.15) | 1359 | 761 | 598 |
| Adolescence poly-E parent-rated & adulthood conduct child-rated | 0.52 (0.46, 0.57) | 0.00 (0.00, 0.02) | 0.09 (0.00, 0.23) | 1358 | 761 | 597 |
| **Nonshared environmental paths** |  |  |  |  |  |  |
| **Poly-E & behaviour problem symptoms** | **e11 (95% CIs)** | **e21 (95% CIs)** | **e22 (95% CIs)** | **N total** | **N MZ** | **N DZ** |
|  |  |  |  |  |  |  |
| Preschool poly-E parent-rated & childhood hyperactivity parent-rated | 0.11 (0.09, 0.13) | 0.01 (0.00, 0.02) | 0.46 (0.41, 0.51) | 1432 | 776 | 656 |
| Preschool poly-E parent-rated & childhood conduct parent-rated | 0.14 (0.12, 0.16) | 0.01 (0.00, 0.02) | 0.29 (0.26, 0.32) | 1388 | 749 | 639 |
| Preschool poly-E parent-rated & childhood emotional problems parent-rated | 0.05 (0.04, 0.05) | 0.00 (0.00, 0.01) | 0.35 (0.32, 0.39) | 1446 | 785 | 661 |
| Preschool poly-E parent-rated & childhood peer problems parent-rated | 0.05 (0.04, 0.06) | 0.01 (0.00, 0.02) | 0.42 (0.37, 0.46) | 1446 | 785 | 661 |
| Preschool poly-E parent-rated & childhood hyperactivity teacher-rated | 0.11 (0.09, 0.13) | 0.00 (0.00, 0.00) | 0.38 (0.34, 0.43) | 1265 | 689 | 576 |
| Preschool poly-E parent-rated & childhood conduct teacher-rated | 0.14 (0.12, 0.17) | 0.00 (0.00, 0.00) | 0.48 (0.43, 0.54) | 1227 | 666 | 561 |
| Preschool poly-E parent-rated & childhood emotional problems teacher-rated | 0.05 (0.04, 0.05) | 0.00 (0.00, 0.01) | 0.42 (0.38, 0.46) | 1273 | 695 | 578 |
| Preschool poly-E parent-rated & childhood peer problems teacher-rated | 0.05 (0.04, 0.06) | 0.00 (0.00, 0.01) | 0.42 (0.37, 0.47) | 1276 | 696 | 580 |
| Preschool poly-E parent-rated & childhood hyperactivity child-rated | 0.11 (0.09, 0.13) | 0.00 (0.00, 0.02) | 0.65 (0.57, 0.73) | 958 | 536 | 422 |
| Preschool poly-E parent-rated & childhood conduct child-rated | 0.14 (0.12, 0.17) | 0.00 (0.00, 0.01) | 0.58 (0.50, 0.66) | 927 | 518 | 409 |
| Preschool poly-E parent-rated & childhood emotional problems child-rated | 0.05 (0.04, 0.05) | 0.00 (0.00, 0.02) | 0.48 (0.42, 0.55) | 964 | 540 | 424 |
| Preschool poly-E parent-rated & childhood peer problems child-rated | 0.05 (0.04, 0.06) | 0.00 (0.00, 0.01) | 0.58 (0.51, 0.66) | 962 | 539 | 423 |
|  |  |  |  |  |  |  |
| Childhood poly-E & adolescence hyperactivity parent-rated | 0.16 (0.13, 0.19) | 0.02 (0.00, 0.03) | 0.27 (0.23, 0.30) | 972 | 541 | 431 |
| Childhood poly-E & adolescence conduct parent-rated | 0.17 (0.14, 0.20) | 0.02 (0.01, 0.03) | 0.21 (0.19, 0.24) | 1004 | 556 | 448 |
| Childhood poly-E & adolescence emotional problems parent-rated | 0.35 (0.30, 0.41) | 0.01 (0.00, 0.02) | 0.36 (0.32, 0.41) | 1043 | 580 | 463 |
| Childhood poly-E & adolescence peer problems parent-rated | 0.32 (0.28, 0.38) | 0.01 (0.00, 0.02) | 0.29 (0.26, 0.33) | 1029 | 575 | 454 |
| Childhood poly-E & adolescence hyperactivity teacher-rated | 0.16 (0.14, 0.20) | 0.00 (0.00, 0.02) | 0.59 (0.52, 0.67) | 570 | 327 | 243 |
| Childhood poly-E & adolescence conduct teacher-rated | 0.18 (0.15, 0.21) | 0.01 (0.00, 0.02) | 0.78 (0.68, 0.90) | 587 | 332 | 255 |
| Childhood poly-E & adolescence emotional problems teacher-rated | 0.35 (0.30, 0.41) | 0.00 (0.00, 0.01) | 0.56 (0.50, 0.63) | 637 | 361 | 276 |
| Childhood poly-E & adolescence peer problems teacher-rated | 0.33 (0.29, 0.39) | 0.00 (0.00, 0.01) | 0.54 (0.48, 0.62) | 627 | 359 | 268 |
| Childhood poly-E & adolescence hyperactivity child-rated | 0.16 (0.14, 0.20) | 0.00 (0.00, 0.02) | 0.56 (0.50, 0.62) | 967 | 539 | 428 |
| Childhood poly-E & adolescence conduct child-rated | 0.17 (0.14, 0.21) | 0.01 (0.00, 0.03) | 0.59 (0.53, 0.65) | 998 | 554 | 444 |
| Childhood poly-E & adolescence emotional problems child-rated | 0.35 (0.30, 0.41) | 0.01 (0.00, 0.03) | 0.48 (0.43, 0.53) | 1115 | 619 | 496 |
| Childhood poly-E & adolescence peer problems child-rated | 0.32 (0.28, 0.38) | 0.00 (0.00, 0.01) | 0.51 (0.46, 0.57) | 1100 | 614 | 486 |
|  |  |  |  |  |  |  |
| Adolescence poly-E parent-rated & adulthood hyperactivity parent-rated | 0.15 (0.13, 0.17) | 0.01 (0.00, 0.01) | 0.44 (0.39, 0.50) | 1625 | 879 | 746 |
| Adolescence poly-E parent-rated & adulthood conduct parent-rated | 0.15 (0.13, 0.17) | 0.00 (0.00, 0.01) | 0.40 (0.36, 0.45) | 1625 | 880 | 745 |
| Adolescence poly-E parent-rated & adulthood hyperactivity child-rated | 0.15 (0.13, 0.17) | 0.00 (0.00, 0.01) | 0.64 (0.58, 0.71) | 1359 | 761 | 598 |
| Adolescence poly-E parent-rated & adulthood conduct child-rated | 0.15 (0.13, 0.17) | 0.00 (0.00, 0.01) | 0.75 (0.67, 0.83) | 1358 | 761 | 597 |
| *Note.* a= genetic path; c= shared environmental path; e= nonshared environmental path; CIs= confidence intervals; N= sample size; MZ= monozygotic twin pairs; DZ= dizygotic twin pairs. |  |  |  |  |  |  |

**Table S****9****.** Genetic, shared and nonshared environmental standardised squared multivariate path estimates for the total sample for the cumulative NSE prediction of behaviour problem symptoms in adulthood from environmental measures in preschool, childhood and adolescence. 

| **Genetic paths** |  |  |  |  |  |  |  |  |  |  |  |  |  |
| --- | --- | --- | --- | --- | --- | --- | --- | --- | --- | --- | --- | --- | --- |
| **Poly-E & behaviour problem symptoms** | **a11 (95% CIs)** | **a21 (95% CIs)** | **a31 (95% CIs)** | **a41 (95% CIs)** | **a22 (95% CIs)** | **a23 (95% CIs)** | **a24 (95% CIs)** | **a33 (95% CIs)** | **a34 (95% CIs)** | **a44 (95% CIs)** | **N total** | **MZ** | **DZ** |
| Preschool, childhood and adolescence poly-E & adulthood hyperactivity parent-rated | 0.30 (0.27, 0.33) | 0.25 (0.19, 0.31) | 0.07 (0.04, 0.10) | 0.03 (0.01, 0.07) | 0.44 (0.37, 0.51) | 0.05 (0.03, 0.08) | 0.07 (0.04, 0.12) | 0.33 (0.29, 0.37) | 0.01 (0.00, 0.04) | 0.42 (0.38, 0.47) | 1441 | 563 | 878 |
| Preschool, childhood and adolescence poly-E & adulthood conduct parent-rated | 0.36 (0.32, 0.40) | 0.33 (0.27, 0.40) | 0.10 (0.07, 0.13) | 0.03 (0.01, 0.07) | 0.30 (0.24, 0.37) | 0.06 (0.03, 0.10) | 0.03 (0.00, 0.08) | 0.28 (0.24, 0.32) | 0.05 (0.02, 0.10) | 0.42 (0.36, 0.46) | 1450 | 561 | 889 |
| Preschool, childhood and adolescence poly-E & adulthood hyperactivity child-rated | 0.30 (0.26, 0.33) | 0.25 (0.19, 0.31) | 0.07 (0.05, 0.10) | 0.00 (0.00, 0.02) | 0.45 (0.38, 0.52) | 0.05 (0.03, 0.09) | 0.04 (0.01, 0.10) | 0.33 (0.29, 0.38) | 0.00 (0.00, 0.02) | 0.28 (0.19, 0.34) | 1057 | 451 | 606 |
| Preschool, childhood and adolescence poly-E & adulthood conduct child-rated | 0.36 (0.32, 0.41) | 0.33 (0.26, 0.40) | 0.09 (0.07, 0.13) | 0.01 (0.00, 0.03) | 0.30 (0.24, 0.37) | 0.06 (0.03, 0.10) | 0.04 (0.00, 0.13) | 0.28 (0.24, 0.32) | 0.01 (0.00, 0.04) | 0.18 (0.06, 0.26) | 1051 | 446 | 605 |
|  |  |  |  |  |  |  |  |  |  |  |  |  |  |
| **Shared environmental paths** |  |  |  |  |  |  |  |  |  |  |  |  |  |
| **E & behaviour problem symptoms** | **c11 (95% CIs)** | **c21 (95% CIs)** | **c31 (95% CIs)** | **c41 (95% CIs)** | **c22 (95% CIs)** | **c23 (95% CIs)** | **c24 (95% CIs)** | **c33 (95% CIs)** | **c34 (95% CIs)** | **c44 (95% CIs)** | **N total** | **MZ** | **DZ** |
|  |  |  |  |  |  |  |  |  |  |  |  |  |  |
| Preschool, childhood and adolescence poly-E & adulthood hyperactivity parent-rated | 0.60 (0.57, 0.63) | 0.04 (0.02, 0.06) | 0.02 (0.01, 0.04) | 0.01 (0.00, 0.02) | 0.12 (0.06, 0.17) | 0.06 (0.02, 0.13) | 0.00 (0.00, 0.01) | 0.32 (0.25, 0.37) | 0.00 (0.00, 0.01) | 0.00 (0.00, 0.01) | 1441 | 563 | 878 |
| Preschool, childhood and adolescence poly-E & adulthood conduct parent-rated | 0.50 (0.47, 0.53) | 0.03 (0.01, 0.05) | 0.04 (0.02, 0.07) | 0.01 (0.00, 0.04) | 0.17 (0.12, 0.22) | 0.14 (0.08, 0.21) | 0.00 (0.00, 0.01) | 0.24 (0.17, 0.30) | 0.00 (0.00, 0.02) | 0.00 (0.00, 0.04) | 1450 | 561 | 889 |
| Preschool, childhood and adolescence poly-E & adulthood hyperactivity child-rated | 0.60 (0.58, 0.63) | 0.04 (0.02, 0.07) | 0.02 (0.01, 0.04) | 0.01 (0.00, 0.03) | 0.11 (0.05, 0.16) | 0.06 (0.01, 0.11) | 0.00 (0.00, 0.03) | 0.32 (0.25, 0.37) | 0.00 (0.00, 0.01) | 0.00 (0.00, 0.05) | 1057 | 451 | 606 |
| Preschool, childhood and adolescence poly-E & adulthood conduct child-rated | 0.50 (0.47, 0.53) | 0.03 (0.01, 0.05) | 0.04 (0.03, 0.07) | 0.01 (0.00, 0.03) | 0.18 (0.13, 0.22) | 0.14 (0.08, 0.20) | 0.01 (0.00, 0.05) | 0.24 (0.17, 0.30) | 0.00 (0.00, 0.02) | 0.00 (0.00, 0.07) | 1051 | 446 | 605 |
|  |  |  |  |  |  |  |  |  |  |  |  |  |  |
| **Nonshared environmental paths** |  |  |  |  |  |  |  |  |  |  |  |  |  |
| **E & behaviour problem symptoms** | **e11 (95% CIs)** | **e21 (95% CIs)** | **e31 (95% CIs)** | **e41 (95% CIs)** | **e22 (95% CIs)** | **e23 (95% CIs)** | **e24 (95% CIs)** | **e33 (95% CIs)** | **e34 (95% CIs)** | **e44 (95% CIs)** | **N total** | **MZ** | **DZ** |
|  |  |  |  |  |  |  |  |  |  |  |  |  |  |
| Preschool, childhood and adolescence poly-E & adulthood hyperactivity parent-rated | 0.10 (0.09, 0.10) | 0.01 (0.00, 0.01) | 0.00 (0.00, 0.00) | 0.01 (0.00, 0.02) | 0.14 (0.12, 0.15) | 0.01 (0.00, 0.01) | 0.01 (0.00, 0.03) | 0.13 (0.12, 0.14) | 0.00 (0.00, 0.00) | 0.40 (0.36, 0.44) | 1441 | 563 | 878 |
| Preschool, childhood and adolescence poly-E & adulthood conduct parent-rated | 0.13 (0.12, 0.14) | 0.01 (0.00, 0.01) | 0.00 (0.00, 0.00) | 0.01 (0.00, 0.01) | 0.14 (0.13, 0.16) | 0.01 (0.01, 0.01) | 0.02 (0.01, 0.03) | 0.13 (0.12, 0.14) | 0.00 (0.00, 0.00) | 0.43 (0.39, 0.46) | 1450 | 561 | 889 |
| Preschool, childhood and adolescence poly-E & adulthood hyperactivity child-rated | 0.10 (0.09, 0.10) | 0.01 (0.00, 0.01) | 0.00 (0.00, 0.00) | 0.00 (0.00, 0.01) | 0.14 (0.12, 0.15) | 0.01 (0.00, 0.01) | 0.00 (0.00, 0.01) | 0.13 (0.12, 0.14) | 0.00 (0.00, 0.00) | 0.65 (0.61, 0.70) | 1057 | 451 | 606 |
| Preschool, childhood and adolescence poly-E & adulthood conduct child-rated | 0.13 (0.12, 0.14) | 0.01 (0.00, 0.01) | 0.00 (0.00, 0.00) | 0.00 (0.00, 0.01) | 0.14 (0.13, 0.16) | 0.01 (0.01, 0.02) | 0.00 (0.00, 0.01) | 0.13 (0.12, 0.14) | 0.00 (0.00, 0.01) | 0.73 (0.68, 0.78) | 1051 | 446 | 605 |
| *Note.* a= genetic path; c= shared environmental path; e= nonshared environmental path; CIs= confidence intervals; N= sample size; MZ= monozygotic twin pairs; DZ= dizygotic twin pairs. |  |  |  |  |  |  |  |  |  |  |  |  |  |

**Supporting references**

Ainley, J., & Bourke, S. (1992). Student views of primary schooling. *Research Papers in Education*, *7*(2), 107–128.

Allegrini, A. G., Karhunen, V., Coleman, J. R., Selzam, S., Rimfeld, K., Von Stumm, S., Pingault, J.-B., & Plomin, R. (2020). Multivariable GE interplay in the prediction of educational achievement. *PLoS Genetics*, *16*(11), e1009153.

Bereiter, C. (1963). *Some persisting dilemmas in the measurement of change. Chapter 1 in Harris, CW (Ed.) Problems in measuring change, Madison, Wisconsin*. University of Wisconsin Press.

Cox, J. L., Holden, J. M., & Sagovsky, R. (1987). Detection of postnatal depression: Development of the 10-item Edinburgh Postnatal Depression Scale. *The British Journal of Psychiatry*, *150*(6), 782–786.

Deater-Deckard, K. (2000). Parenting and child behavioral adjustment in early childhood: A quantitative genetic approach to studying family processes. *Child Development*, *71*(2), 468–484.

Deater–Deckard, K., Dodge, K. A., Bates, J. E., & Pettit, G. S. (1998). Multiple risk factors in the development of externalizing behavior problems: Group and individual differences. *Development and Psychopathology*, *10*(3), 469–493.

Gidziela, A., Rimfeld, K., Malanchini, M., Allegrini, A. G., McMillan, A., Selzam, S., Ronald, A., Viding, E., Von Stumm, S., & Eley, T. C. (2021). Using DNA to predict behaviour problems from preschool to adulthood. *Journal of Child Psychology and Psychiatry*.

Knopik, V. S., Neiderhiser, J. M., DeFries, J. C., & Plomin, R. (2017). *Behavioral genetics*. Worth Publishers, Macmillan Learning.

Matheny Jr, A. P., Wachs, T. D., Ludwig, J. L., & Phillips, K. (1995). Bringing order out of chaos: Psychometric characteristics of the confusion, hubbub, and order scale. *Journal of Applied Developmental Psychology*, *16*(3), 429–444.

Neale, M. C., Hunter, M. D., Pritikin, J. N., Zahery, M., Brick, T. R., Kirkpatrick, R. M., Estabrook, R., Bates, T. C., Maes, H. H., & Boker, S. M. (2016). OpenMx 2.0: Extended structural equation and statistical modeling. *Psychometrika*, *81*(2), 535–549.

Price, T. S., Freeman, B., Craig, I., Petrill, S. A., Ebersole, L., & Plomin, R. (2000). Infant zygosity can be assigned by parental report questionnaire data. *Twin Research and Human Genetics*, *3*(3), 129–133.

Rijsdijk, F. V., & Sham, P. C. (2002). Analytic approaches to twin data using structural equation models. *Briefings in Bioinformatics*, *3*(2), 119–133.

Rimfeld, K., Malanchini, M., Spargo, T., Spickernell, G., Selzam, S., McMillan, A., Dale, P. S., Eley, T. C., & Plomin, R. (2019). Twins early development study: A genetically sensitive investigation into behavioral and cognitive development from infancy to emerging adulthood. *Twin Research and Human Genetics*, *22*(6), 508–513.

Rovine, M. J. (1994). Estimating nonshared environment using sibling discrepancy scores. *Separate Social Worlds of Siblings: The Impact of Nonshared Environment on Development*, *3*, 33–61.

R Core Team (2021). R: A language and environment for statistical computing. R Foundation for Statistical Computing, Vienna, Austria. URL https://www.R-project.org/.

Turkheimer, E., & Waldron, M. (2000). Nonshared environment: A theoretical, methodological, and quantitative review. *Psychological Bulletin*, *126*(1), 78.

Vitaro, F., Brendgen, M., & Arseneault, L. (2009). The discordant MZ-twin method: One step closer to the holy grail of causality. *International Journal of Behavioral Development*, *33*(4), 376–382. https://doi.org/10.1177/0165025409340805
